# Metabolomics as an Approach to Characterise the Contrasting Roles of CCR5 in the Presence and Absence of Disease

**DOI:** 10.3390/ijms21041472

**Published:** 2020-02-21

**Authors:** Anandi Rautenbach, Aurelia A. Williams

**Affiliations:** Human Metabolomics, North-West University, Private Bag X6001, Box 269, Potchefstroom 2531, South Africa

**Keywords:** CCR5, immunology, metabolism, metabolomics, immunometabolism, disease

## Abstract

Chemokine receptors such as C-C chemokine receptor 5 (CCR5) are activated through interaction with their ligands and are well known for their role in chemotaxis and signal transduction. While serving these roles, cellular responses are effected, hence the immune function of these molecules is established. Given the role of CCR5 in immune function and that the immune and metabolic systems are interlinked, subsequent immune-directed changes should be measurable at a metabolic level. Numerous investigations have reported on metabolic changes associated with CCR5 status in the presence of disease, so as to understand whether specific CCR5 genotypes, frequency and/or levels offer protection to the host or not. However, these metabolic changes were recorded using older conventional techniques. Depending on certain factors such as the disease model, the geography of the samples and/or the ethnic group under study, the role of CCR5 in disease differs. In addition, little is known about CCR5’s role in the absence of an enhanced inflammatory state, such as when infection persists. Metabolomics is defined as the study of metabolites and informs on metabolic changes within living organisms as induced by various stimuli, such as the interaction of CCR5 with its ligand. Since metabolomics reflects the underlying biochemical activity and state of cells/tissues, this review proposes it as a tool to clarify the contrasting roles of CCR5.

## 1. Introduction

Chemokines are small proteins with molecular weights ranging from 7 to 15 kilodalton (kDa) [1]. These molecules form part of the cytokine family. Cytokines are small proteins secreted by cells of the immune system and act as signaling molecules which regulate the interactions between cells. Cytokines have the ability to maintain the balance between humoral and cell-mediated immune (CMI) responses. Their activities are often redundant since different cytokines have the ability to stimulate similar functions. With respect to the immune system, chemokines may also exert different/dual functions. Chemokines can be pro-inflammatory stimulating inflammation and the production of more chemokines, or anti-inflammatory inhibiting inflammatory reactions as well as the production and/or release of chemokines. Chemokines thus function as chemotactic factors, directing the response of cells to chemical stimuli [2]. More specifically, chemokines are involved in the trafficking of a variety of leukocytes to a site of infection or tissue damage. In addition to chemotaxis, chemokines play a role in several other biological processes such as angiogenesis and hematopoiesis, as well as aid in embryonic development and metastasis [3,4].

The chemotactic function of chemokines is facilitated through the presence of chemokine receptors which occurs on the surface of cells. These receptors are naturally expressed on most cells but are especially produced during diseased states [5,6]. Chemokines and their receptors are found to play a pivotal role in many diseases. One of the most studied proteins in this regard is C-C chemokine receptor type 5 (CCR5). The CCR5 gene is located on the short arm position 21 on chromosome 3, denoted as 3p21 [7]. The CCR5 gene undergoes translation, resulting in the CCR5 protein which has a molecular weight of 40.6 kDa [8], comprising 352 amino acids [9]. This G-protein coupled chemokine receptor is expressed on numerous cell types. The cell types include cells of the immune system, namely, dendritic cells, macrophages as well as memory T-cells; epithelial cells, endothelial cells; fibroblasts, vascular smooth muscle and cells of the central nervous system such as microglia, astrocytes and neurons [10]. Several ligands for this chemokine receptor exist including: macrophage inflammatory protein one alpha (MIP1-α) [11], macrophage inflammatory protein one beta (MIP1-β), regulated upon activation, normal T-cell expressed and secreted (RANTES) which is also known as C-C chemokine ligand 5 (CCL5) and monocyte chemotactic protein two (MCP-2) [7,8,12,13].

The immune system consists of several defence mechanisms against any foreign substance. During the innate immune response which involves chemokine production, inflammation forms a key part of the response. CCR5 plays a key role during inflammation. More specifically, CCR5 ligands such as CCL5 are released at sites of infection or tissue damage in order to activate effector cells. These effector cells release chemokines for further signal amplification [14,15]. When receptors interact with a specific ligand to perform its specific function, the protein receptor undergoes a conformational change. The interaction between chemokine and receptor thus attracts immune cells to affected tissue/organ sites and allows the receptor to send biological signals to other parts of the body effecting a cellular response [16]. Other changes include the improved movement of the cell(s) and enhanced secretion of lysosomal enzymes involved in the degradation of waste products.

The adaptive immune response comprising antibody production and CMI responses provides protection at a later stage of an infection and is able to efficiently detect any known pathogen since it establishes a state of memory. During CMI, CD4^+^ T-helper cells regulate the immune response as well as the activation of other immune cells. The cytotoxic CD8^+^ T-cells eliminate target cells that express a foreign antigen. The expression of CCR5 on CD8^+^ T-cells is especially upregulated during inflammation [17]. This process increases the chance of CD8^+^ T-cells to encounter antigen-specific cells. Therefore, in addition to the role of CCR5 in the innate immune response, CCR5 expression also has an influence on the adaptive immune response. Cells are often characterised as either T-helper type 1 (Th1) or T-helper type 2 (Th2) depending on the immune response and the cytokine profile they induce. Th2 immune responses usually counteract the effect of the Th1 response [18]. Ideally, the Th1 and Th2 immune responses should be balanced to effectively eliminate pathogens [19]. The expression of CCR5 is limited to Th1 cells [20]. The amount of CCR5 present on the cell’s surface can thus influence T-cell immune responses and ultimately alter the balance of Th1 and Th2 immune responses to impact disease outcome.

In addition to the role of CCR5 in immune function, CCR5 also affects metabolism namely, ligands/chemokines such as CCL5 through interaction with their CCR5 receptors, mediates metabolic change [21,22] inducing a hypermetabolic state in the affected system. Studies that have reported on CCR5-influenced metabolic changes in the presence of disease include, for example, that of Gao [22] as well as Bonfa [23], where metabolic changes during cancer and *Toxoplasma Gondii* infection were investigated, respectively. In a previous study done on cancer cells, Gao et al. [22] showed that CCL5 activated CCR5 and that this activation impacted on glycolysis as well as adenosine triphosphate (ATP) production. Cancer cells, when treated with CCL5, showed increased expression of glucose transporters which increased glucose uptake and therefore glycolysis. Metabolic analysis revealed that cancer cells treated with CCL5 reflected increased anabolic pathways, which are responsible for synthesizing complex molecules. The increase in some metabolic pathways ultimately resulted in increased ATP production [22]. Bonfa and co-workers [23] used a CCR5-deficient mouse model and reported decreased levels of several inflammation-related cytokines and transcriptional factors during *Toxoplasma Gondii* infection. In addition, they reported that CCR5 deficiency resulted in elevated serum triglycerides and finally liver metabolic dysfunction [23]. Bing and co-workers [24] used a rat model and reported on the expression and function of CCR5 during the course of type II diabetes mellitus. The authors used an antibody to block CCR5 function and measured metabolic changes. Diabetic mice presented increased levels of low density lipoprotein (LDL) and increased fasting blood triglyceride levels. Based on these conventional indicators (i.e., spectrometric high density lipoprotein (HDL) and LDL), the group was able to show that blocking CCR5 function improved diabetes in their model [24].

It is established that there is a clear relationship between immunology and metabolism. This has led to the term immunometabolism which aims to clarify amongst other factors the involvement of inflammation in the pathogenesis of several metabolic disorders [25]. Furthermore, it also describes different metabolic factors that have an influence on the regulation of several different immune cell functions [26]. For example, Ma et al. [27] showed that optimal T-cell expansion is dependent on serine, a metabolite vital for purine metabolism [27]. Pan et al. [28] showed that some memory T-cells are dependent on fatty acids for eliciting effective immune responses [28]. Although these studies are not CCR5-specific, they clearly highlight the immuno-metabolic concept. Given the role of CCR5 in immune function, subsequent changes linked to the chemokine receptor should thus be measurable at the metabolic level. Although literature highlighting the role of CCR5 in immune system function as well as metabolism is available, the latter is very limited and/or recorded using older conventional systems as in the study by Bing and colleagues [24]. Minimal information is also available on CCR5’s role in the absence of an enhanced inflammatory state such as when infection persists.

The functionality of CCR5 is compromised when the gene coding for CCR5 is subject to mutations. One such mutation involves the 32-base pair deletion, CCR5∆32, which is present in about 10% of the Caucasian population [29]. This 32 bp deletion from nucleotide position 794 to 825 results in a frameshift mutation, an altered codon reading frame and a dysfunctional translation mechanism. The entire structure of the gene is altered yielding a mutated protein composed of only 215 instead of 352 amino acids. The last three transmembrane domains of CCR5 along with regions critical for cellular processes, such as G-protein interactions and signal transduction, are thus absent in the mutated protein. This has implications for immune and subsequent metabolic processes. The end result is altered expression and function of the receptor [30,31]. Consequently, several CCR5 genotypes exist in this regard i.e., individuals can present with a homozygous 32 bp deletion genotype presenting no CCR5 thus ligands and/or other chemokines cannot bind. Alternatively, individuals can present with a wild type (WT) homozygous genotype where normal levels of CCR5 is expressed or a heterozygous genotype where minimal CCR5 is expressed. The CCR5 genotype can therefore be directly correlated to the cell surface expression of CCR5, also noted as CCR5 levels. Individuals can thus present with no, low or high CCR5 levels depending on the genotype. In more recent studies, authors categorised cohorts according to their haplotype. Haplotype takes into account the different polymorphisms on the same chromosome and are categorised as either protective or non-protective [32].

In the case of diseases where CCR5 is involved in pathogen entry, individuals presenting with the homozygous 32 bp deletion will not express CCR5, while individuals with the heterozygous 32 bp deletion will express lower amounts of CCR5 and are therefore characterised as having a protective phenotype. In contrast, individuals presenting the WT homozygous genotype, express higher levels of CCR5 and are thus characterised as having a non-protective phenotype. A protective haplotype will therefore take polymorphisms leading to lower CCR5 expression into account, while non-protective haplotypes are associated with increased CCR5. More recently, Jaumdally and colleagues [33] in their 2017 study investigated inflammation and T-cell activation in the plasma of uninfected participants, exposed and unexposed to human immunodeficiency virus type 1(HIV-1), the causative agent of acquired immunodeficiency syndrome (AIDS). Exposed uninfected individuals had lower frequencies of CCR5-positive (CCR5^+^) immune cells (protective against infection) than their unexposed counterparts. These individuals when grouped thus expressed protective CCR5 haplotypes and presented with reduced inflammatory cytokines. The data obtained by Jaumdally and co-workers [33] provides important biological information regarding samples presenting differential CCR5 status even before actual infection. The terms used to describe CCR5 (i.e., genotype, levels, haplotype, etc.) are thus all interlinked.

Much of the attention and clinical relevance related to CCR5 has centred on its role as co-receptor, facilitating viral entry during HIV-1 and other infections. The role of CCR5 in HIV-1 was established when authors showed that CCR5 on the surface of CD4^+^ T-cells has a direct correlation with infectability/viral levels [34]. As such, the role of the 32 bp deletion is increasingly investigated in context to HIV-1 disease susceptibility [35] and progression [36,37]. As a result of the failed expression of CCR5, the 32 bp deletion is associated with resistance against certain infections, such as HIV-1 [38], while others show the mutation to facilitate disease progression [35], mainly due to failed chemotactic function. Section 2 of this article details more of the contradictory roles of CCR5 in disease.

The therapeutic role of blocking CCR5 has also become of increased research interest. In the case of diseases where CCR5 is needed for viral entry into the cell, such as with HIV-1 infection, a CCR5 antagonist inhibits viral entry into the cell and therefore limits disease development and progression. Maraviroc is a common CCR5 antagonist used to inhibit HIV-1 entry. In addition, maraviroc is also shown to be successful at attenuating disease severity in several other conditions [39]. While the mini-review by Vangelista and co-workers [40] also reports on the role of CCR5 in a few diseases, the authors focus on the therapeutic benefit of blocking CCR5 function, biochemically or through molecular therapies.

With respect to CCR5, the 32 bp deletion occurring in Caucasians is however not the only mutation known to alter the molecule’s expression and/or function. A novel 24-base pair deletion with phenotypic effects similar to that of CCR5∆32 was recently reported in the coding region of CCR5 in African individuals [41]. Numerous other genetic mutations within the CCR5 gene structure have been studied and linked to disease progression [42,43,44,45,46]. A full discussion of other mutations within CCR5 lies beyond the scope of this article. Further discussions will thus only focus on the 32 bp deletion given its current clinical relevance and media attention received.

In 2009 Hütter and co-workers [47] reported on the first patient to be cured of HIV-1 infection. The patient, commonly known as the “Berlin Patient”, underwent a hematopoietic stem cell transplantation as part of his treatment for leukaemia. The protective homozygous CCR5∆32 genotype was carried by the stem cell donor [47]. In subsequent years there were reports that yet another patient maintained remission of HIV-1 infection. This patient, known as the “London patient”, underwent two allogenic hematopoietic stem cell transplants using a donor with a homozygous CCR5∆32 genotype. Complete remission was observed six months after the stem cell transplant [48]. At the end of 2018, CCR5 made the news once again when a Chinese geneticist, He Jiankui, engineered HIV-1 resistant babies. Clustered regularly interspaced short palindromic repeats (CRISPR) are short DNA sequences present in living organisms. Scientists recently started using Cas9, a protein with the ability to cut DNA strands, in conjunction with these CRISPR DNA sequences to alter an organism’s genetic material. It is reported that Jiankui used CRISPR-Cas9 to generate genetically modified embryos carrying the CCR5∆32 mutation. This results in an individual with cells devoid of functional CCR5 on their surface [49]. Whilst this mutation is generally protective against HIV-1 infection, the long-term effects of such an experiment are not yet known. While some researchers report individuals carrying the 32 bp deletion to live a normal life [40], others report on the negative effects of the mutation (i.e., that it is associated with increased susceptibility to various common diseases and increased mortality). Conclusions regarding the negative effects of CCR5∆32 were deduced using data from the United Kingdom (UK) Biobank [50]. The article has since been retracted due to genotyping calling bias in the dataset used. Using the same dataset as the preceding article, Maier et al. [51] reported that the homozygous CCR5∆32 does not influence the lifespan of individuals harbouring the homozygous CCR5∆32 genotype [51]. This area of research thus remains open for further investigation.

In addition to mutations, the expression of receptors and their ligands is influenced by disease. Cancer, a condition in which cells grow abnormally, presents with the increased expression of CCL5 and CCR5. Furthermore, receptor expression is also influenced by epigenetic modifications such as DNA methylation [52]. These modifications alter the activity of DNA without changing the actual DNA sequence. In the case of CCR5, DNA methylation is inversely related to the amount of CCR5 present on the surface of T-cells. When T-cells are activated (such as during infection), demethylation occurs resulting in upregulation of CCR5 expression.

**Figure 1:**
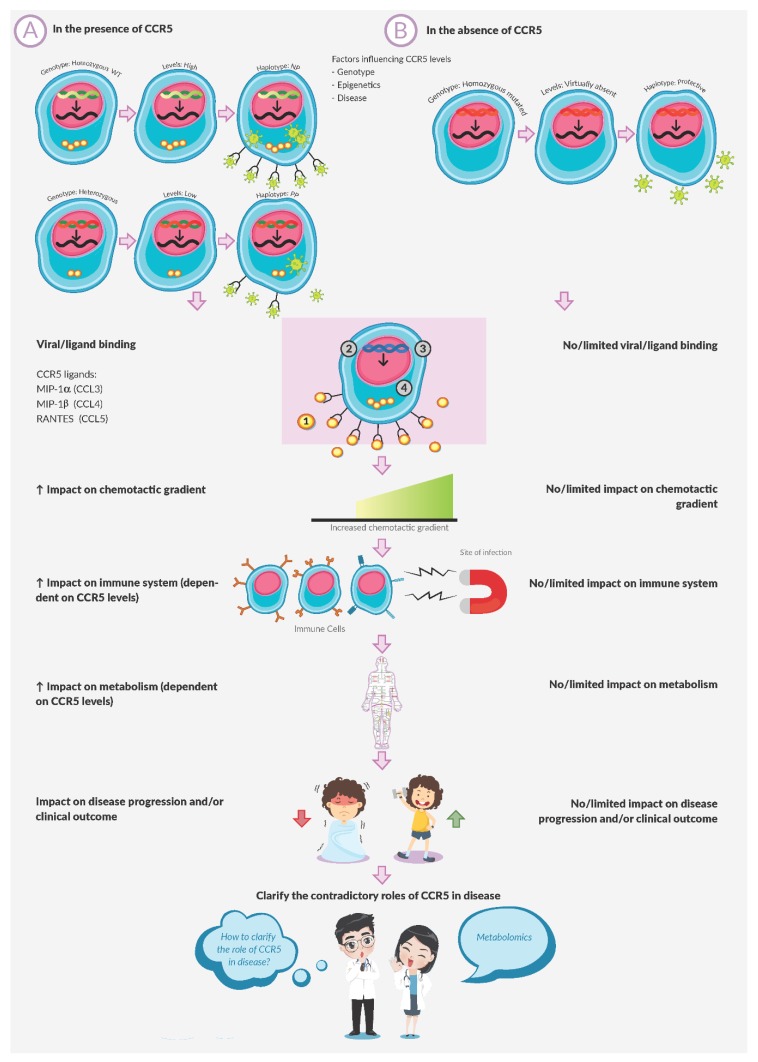
Graphical display of the cascading effect of CCR5 on signal transduction, chemotaxis, immune function and metabolism, all of which ultimately impact disease progression and/or clinical outcome. The cascading effect presents an opportunity to specifically measure the immuno-metabolic changes through metabolomics. Several factors influence CCR5 status. We depict scenarios for when (**A**) CCR5 is present (high and low levels) and (**B**) absent. In the presence of CCR5, (1) viral/ligand binding occurs and (2) induces a cell signaling cascade that causes (3) gene activation leading to (4) a protein product and/or cellular response. (**B**) Lack of viral/ligand binding due to the absence of CCR5 limits the signaling cascade and its subsequent processes. Abbreviations: CCR5—C-C chemokine receptor 5; CCL3—C-C chemokine ligand 3; CCL4—C-C chemokine ligand 4; CCL5—C-C chemokine ligand 5; MIP1-α— macrophage inflammatory protein one alpha; MIP1-β—macrophage inflammatory protein one beta; NP—non-protective; PP—partially protective; RANTES—regulated upon activation, normal T-cell expressed and secreted; WT—wild type.

Considering the role of chemokines, it is anticipated that the differential expression patterns of chemokine receptors and their respective ligands may play a role in inflammation and impact the immune response and metabolism to ultimately influence disease progression and/or clinical outcome. The metabolic change(s) in particular should then be measurable using metabolomics. The role of CCR5 in this regard, which is also the essence of this review, is summarised in Figure 1. Major diseases in which the role of CCR5 is investigated is reviewed in Section 2 and summarised in Table 1. In the table, a summary of the disease model used to investigate the role of CCR5 is presented, together with the primary assays performed and parameters measured. Where immune and metabolic parameters were measured these are highlighted in the text to further justify the metabolomics approach proposed here. We realise that not all studies are reported in this article and while some of the reported studies overlap with information reported by Klein [53] and Vangelista and co-workers [40], we however highlight a broader range of conditions to show the contrasting roles of CCR5 therein with possible reasons for the discrepancies. Given CCR5’s immuno-metabolic effects, metabolomics is proposed as an approach with which to clarify the receptor’s role.

## 2. The Role of CCR5 in Disease

### 2.1. Cardiovascular Diseases

The cardiovascular system comprises of the heart, surrounding arteries and blood vessels. CCR5 is present on the surface of leukocytes which are transported through the body via circulating blood. Several diseases of the cardiovascular system exist and it is well known that inflammatory processes play a role during such conditions. As a result, chemokines and chemokine receptors are implicated in cardiovascular diseases (CVD). The signalling molecules in the immune system are responsible for maintaining homeostasis and initiating an immune response when necessary. However, continuous inflammatory responses during the diseased state can influence disease progression [54]. Therefore, since inflammatory processes play a role in cardiovascular diseases, CCR5 may be involved in cardiovascular disease progression. Several studies have therefore been conducted to assess the role of CCR5 in diseases related to the cardiovascular system.

Atherosclerosis is caused by a build-up of cholesterol and fatty deposits on the inner walls of the arteries which impedes blood flow, resulting in a variety of conditions [55]. Atherosclerosis can be categorised as an inflammatory disease of the cardiovascular system due to the cellular responses present in atherosclerotic lesions [56]. The inflammatory response is primarily mediated by macrophages as well as specific T lymphocytes involved in the specific stage of atherosclerosis disease [57]. Activation of the lymphocytes results in the release of several inflammatory mediators such as chemokines and cytokines [58]. The chemokines are involved in the migration and accumulation of macrophages within fatty streaks, lesions that develop into fibrous plaque. During the development of atherosclerotic plaques, several immune cells are recruited continuously which is a characteristic of the chronic inflammatory response [59]. An example of a chemokine that is expressed in large quantities in atherosclerotic plaques is monocyte chemoattractant protein-1 (MCP-1) [56]. Readers with an interest in this chemokine are directed to the article of Lin and co-workers [60] which provides a detailed review of MCP-1 [60].

A meta-analysis was performed by Zhang and colleagues [61] regarding the association of CCR5∆32 and susceptibility to atherosclerotic disease. The authors combined 13 case-controlled studies comprising various ethnic cohorts and established that there was no association between CCR5∆32 and atherosclerotic disease susceptibility. Surprisingly, further subgroup analysis revealed an increase in the frequency of CCR5∆32 in Asian patients and a significant association of the mutation with increased susceptibility to atherosclerotic disease [61]. Atherosclerosis can also result in a variety of sub-conditions, explained in the sections that follow.

Atherosclerosis results in coronary artery disease (CAD) which develops when coronary arteries become narrow or in some cases blocked. Several studies report on the role of CCR5Δ32 in CVD. We highlight a few of these studies which showed CCR5Δ32 to (a) be a risk factor for CVD, (b) offer protection against CVD and (c) have no impact on CVD. Several studies using blood found that, although there was mostly no difference in the frequencies of CCR5Δ32 among patients and controls [62,63,64,65], the 32 bp deletion may still be a risk factor for the development of atherosclerosis [62,66]. In the study conducted by Pai and co-workers [62], seven polymorphisms within chemokine receptors CCR5 and CCR2 were studied. The authors found no difference in allele distributions between cases and controls. None of the haplotypes were associated with the risk of coronary heart disease. The authors did not observe the homozygous CCR5∆32 genotype among patients when cases were stratified according to age and thus found CCR5∆32 to be associated with early age of onset. In addition, the authors measured immune and metabolic parameters. Inflammatory markers such as C-reactive protein (CRP) and cytokines were measured using an immunoturbidimetric high sensitivity assay and the enzyme-linked immunosorbent assay (ELISA), respectively while cholesterol was measured using standard protocols. Coronary heart disease patients presented with increased inflammatory markers and lipids and decreased HDL-C when compared to controls [62]. In the study conducted by Sharda and co-workers [66], the authors determined CCR5Δ32 genotypes in a North Indian cohort using the polymerase chain reaction (PCR). The authors reported an increased frequency of heterozygous CCR5Δ32 genotype among patients when compared to controls but the increase was statistically insignificant (hence the heterozygous superscript is not shown for [66], Table 1). Furthermore, the homozygous CCR5Δ32 genotype was absent in the cohort. Metabolic analysis of the patients’ blood showed significant increases in total cholesterol, triglycerides, LDL and apolipoprotein B compared to controls. The authors did not specify the methods used to determine these parameters. Based on the higher frequency of the mutation within patients, the authors suggested that it might be associated with risk of developing CAD [66].

Some studies found CCR5Δ32 to be associated with protection against CVD-related conditions. Hyde and co-authors [63] used blood samples from two populations enrolled in a cardiovascular prevention trial. The authors investigated possible genetic associations with circulating lipid levels and ultimately the influence of genetic polymorphisms such as CCR5Δ32 on cardiovascular disease. While there was no association between the heterozygous CCR5Δ32 genotype with lipid levels and cardiovascular disease, the authors did show an association between the homozygous CCR5Δ32 genotype, increased high-density lipoprotein cholesterol (HDL-C) and decreased triglycerides (TG) levels, respectively. HDL-C is known to act as a scavenger to aid in the removal of low-density lipoprotein cholesterol (LDL-C) from arteries. TG is linked to the fatty build-up present in the arteries of CAD patients. Therefore, the increased HDL-C and decreased TG levels present in homozygous CCR5Δ32 carriers are associated with protection against CAD [63]. In a study by Afzal and co-workers [67], the role of CCR5Δ32 as a risk factor for CVD in a Bruneck population was investigated. The authors used PCR to determine the CCR5 genotypes of CVD patients. In addition, the authors determined the levels of several inflammatory markers and HDL using immune and enzymatic assays, respectively. Furthermore, an ultrasound was used to determine the thickness of arteries. The authors reported both homozygous and heterozygous CCR5Δ32 to be associated with decreased inflammatory markers, incident of CVD and risk of death. The authors concluded that both homozygous and heterozygous CCR5Δ32 genotypes offer some protection against CVD [67]. Lassner and co-workers [68] determined the CCR5Δ32 frequency in patients compared to controls and investigated the mortality rates of the cohort. Genotypes were determined using PCR. Although there was no difference in frequencies of CCR5Δ32 between the two groups, the study found that the patient cohort carrying either the homozygous or heterozygous CCR5Δ32 mutation presented decreased mortality rates over a six-year period compared to healthy controls, and thus associated CCR5Δ32 with protection against non-ischemic cardiomyopathy (CM) [68]. Szalai and co-workers [69] investigated various CCR5 related polymorphisms in CAD patients and healthy controls. The authors genotyped all individuals and determined lipoprotein(a) levels using enzymatic methods. While the heterozygous CCR5∆32 genotype had no effect on CAD susceptibility, the absence of homozygous CCR5∆32 genotypes in the patient group led the authors to suggest a protective role for homozygous CCR5∆32 in the context of CAD [69].

In contrast, some studies found that CCR5Δ32 did not have an effect on CAD susceptibility. Apostolakis and co-workers [64] investigated the role of several polymorphisms within the chemokine and chemokine receptor genes and their involvement in susceptibility to CAD. The genotypes of all CAD patients and healthy controls participating in the study was determined by either PCR alone or PCR combined with restriction fragment length polymorphism (RFLP). The DNA purity was determined using spectroscopy. Individuals carrying the homozygous CCR5Δ32 genotype were absent in this cohort. The authors reported no significant differences in allele and genotype frequencies among CAD patients and controls and therefore no association of CCR5Δ32 with CAD [64]. The association of CCR5Δ32 with CAD was investigated by Simeoni and co-authors [65]. Individuals’ samples were genotyped using PCR/RFLP methods and individuals were divided into CAD cases according to coronary angiography results. CRP was measured using a nephelometric immunoassay while standard metabolic parameters defining CAD were reported. CRP was increased among patients, but not associated with CCR5Δ32. Homozygous CCR5Δ32, heterozygous CCR5Δ32 and homozygous WT genotypes were present among patients and controls. The allelic and genotypic frequencies did not differ significantly between patients and controls. The authors thus concluded that CCR5Δ32 is not associated with CAD susceptibility [65]. A meta-analysis comprising 10 studies that focused on CCR5Δ32 was performed by Wang and colleagues [70]. The authors found no significant association of the polymorphisms in the CCR5 and CCL5 genes with CAD risk. Four of these studies associated the CCR5Δ32 mutation with protection against CAD, while three found no association between CCR5Δ32 and CAD. Overall, it appears that CCR5Δ32 is not associated with CAD risk. Other studies used in the meta-analysis focused on polymorphisms within the CCL5 gene [70].

Myocardial infarction (MI), more commonly known as a heart attack, occurs when the blood flow to a section of the heart is decreased, resulting in damaged heart muscles. During MI, the chemokine MCP-1 is involved in post infarction remodelling, a process that refers to changes in the structure and function of the heart due to loss of myocardium. Elevated levels of MCP-1 are present in MI patients and are associated with disease prognosis [71]. The CCR5∆32 mutation is associated with protective effects against MI. In an investigation by Balistreri and co-authors [72], Sicilian patients and healthy controls were genotyped using blood samples. The authors reported a significant increase of allelic and genotypic frequencies of CCR5Δ32 in controls compared to patients. Based on these results, the authors suggested a protective role for CCR5Δ32 against acute MI [72]. In a study performed by Gonzalez et al. [73], CCR5Δ32 was associated with age of onset of MI. Genotypes were determined using PCR. The authors reported absence of the homozygous mutation in patients <55 years and increased CCR5Δ32 allelic frequencies in controls when compared to patients. In addition, CCR5Δ32 was increased in patients >60 years compared to patients <55 years. Therefore, the authors suggested a possible protective effect of CCR5Δ32 against early MI [73]. CCR5Δ32 was also associated with increased risk for MI in a Turkish population. In an investigation performed by Karaali and co-workers [74], genotypes were determined using PCR/RFLP. The authors showed increased allelic frequencies of homozygous and heterozygous CCR5Δ32 in patients compared to controls. A significant increase in cholesterol and LDL-C levels were also reported in patients compared to controls. Consequently, the authors concluded that the CCR5Δ32 genotypes were associated with an increased risk of developing MI [74].

Atherosclerosis can cause carotid stenosis when plaque build-up within the artery carrying blood to the brain, occurs. One study determined the role of various variants within chemokines and chemokine receptors as a risk factor for internal carotid artery stenosis (ICAS). Ghilardi and co-workers [75] obtained blood samples from an Italian cohort and used PCR to genotype individuals for various polymorphisms. Regarding CCR5∆32, the authors found that there were no significant differences in genotypic and allelic frequencies of CCR5∆32 in patient and control groups. Therefore, the authors concluded that CCR5∆32 does not have an effect on ICAS development [75]. 

Hypertension refers to the condition in which blood exerts force against the walls of blood vessels causing blood pressure to increase to unhealthy levels. The link between the CCR5∆32 variant and hypertension formed the central focus of a study by Mettimano and co-workers [76] in which the authors investigated polymorphisms within the CCR5 and CCR2 genes in hypertensive patients, respectively. For this analysis, blood was used and the cohorts included Caucasians due to the increased prevalence of the CCR5∆32 mutation in this group. The authors reported increased allelic frequency of CCR5∆32 in the patient group and therefore showed a clear association between CCR5∆32 and hypertension [76]. Unlike Mettimano, Zhang and co-authors [77] could not confirm this association when they conducted a larger study in 2006 comprising subjects from the United States and Poland. The authors reported that the allelic frequency of CCR5∆32 was the same in the patient and control groups. In addition, CCR5∆32 did not have an influence on blood pressure and did not show an association with hypertension in any age group [77].

### 2.2. Diseases of the Nervous System

The central nervous system is mainly comprised of the brain, spinal cord and peripheral nerves. The overall health of the central nervous system is conserved by a specialised population of cells. These cells include microglial cells which are involved in the removal of damaged neurons, astrocytes which are associated with neuronal synapses and the regulation of transmission of electrical impulses within the brain, as well as specialised cells with the ability to transmit signals to the rest of the body known as neuronal cells [78]. These cells continuously produce chemokines in order to maintain a state of homeostasis within the brain. Both chemokines and their receptors influence the migration and differentiation of these specialised cells. Inflammatory mediators have the ability to enhance the chemokine receptor–ligand interaction. Therefore, chemokines and their receptors are associated with a variety of inflammatory conditions [79], including nervous system diseases such as Alzheimer’s disease (AD), Parkinson’s disease (PD), multiple sclerosis (ms), tumour progression, stroke and AIDS-associated dementia [79], to name a few.

Alzheimer’s disease is a chronic neurodegenerative disease and is the most common form of dementia. It is a progressive condition in which brain cells degenerate [80]. AD patients usually present with beta amyloid plaques, a protein fragment which is eliminated under normal conditions, but forms insoluble plaques within the brain of AD patients. The formation of these plaques result in neuron degeneration. Disease progression is enhanced by pro-inflammatory processes. The chemokines and receptors present in the central nervous system play a pivotal role in neuro-inflammation. The expression of cytokines and other inflammatory mediators is upregulated during the neuro-inflammatory response [81], showing an association with pathological changes seen in AD patients [82]. Similarly, chemokine receptors are also involved in the pathophysiology of AD. It was reported that CCR5 and CCR2 levels/expression increase in AD patients compared to controls. Upregulation of chemokine receptors and their ligands were found in AD brain and that may contribute to plaque-associated inflammation and neurodegeneration [79]. Given the role of CCR5 in the immune system, it is expected that CCR5 deficiency is associated with protection against inflammatory diseases. CCR5 deficiency however can result in activation of the astrocytes as well as amyloid-beta deposits. One of the main consequences of amyloid-beta deposits include memory dysfunction due to enhanced CCR2 expression [83]. Several studies were conducted regarding CCR5∆32 and its association with AD. Combarros and co-workers [84] conducted a study and reported on the lack of association of CCR5∆32 with AD. Combarros et al. [84] used PCR to determine the genotypes of their Spanish cohort and did not find a significant difference in the allelic or genotypic frequencies of CCR5∆32 between AD patients and controls. In addition, the authors reported that CCR5∆32 did not influence the age of onset of AD [84]. Balistreri and co-workers [85] investigated the possible protective role of CCR5∆32 in AD using blood samples from an Italian cohort. The control group consisted of elderly individuals that did not present with dementia. The authors did not find significant differences in the allelic distribution and frequency of CCR5Δ32 between AD patients and controls. In addition, the authors showed neither gender nor disease progression to be associated with CCR5∆32 [85]. These results confirm findings of a previous study done by Galimberti and co-workers [86] who also used an Italian cohort. The authors obtained blood samples and determined the CCR5∆32 genetic distributions in patients and healthy controls. The authors found no difference in genotypic frequencies among patients and controls; CCR5∆32 frequencies did not differ when cases were stratified by gender or age of onset [86]. Similar results were obtained by another study that investigated the role of CCR5 in AD using an Iranian cohort. The CCR5∆32 frequency was determined post PCR and RFLP genotyping of blood samples. The authors reported a lack of association of CCR5∆32 with AD due to similar CCR5∆32 genotypic distributions in patients and controls [87]. Collectively, these studies showed a lack of association of CCR5∆32 with AD susceptibility, progression and age of onset. However, other mutations linked to inflammation might play a role in AD. Jorda et al. [88] investigated the role of the chemokine system in AD using a specific mouse model generated for this condition. Reverse transcription-PCR (RT-PCR) and Western blots were used to determine the expression of CCR5 and various chemokines. The authors observed that the AD-representing mice showed decreased CCR5 levels compared to the WT mice. In this model the decreased CCR5 suggests a possible increase in astrocytes which is important for transmission of electrical impulses in the brain. The authors concluded that the varying chemokine levels could be used to explain the inflammation seen in the brains of AD patients [88]. An in-depth review article regarding CCR5 and AD was published by Li and Zhu [89].

Parkinson’s disease is a progressive nervous system disorder that affects nerve cells within the brain which are responsible for the production of dopamine. Dopamine, a neurotransmitter, is a chemical that transmits signals to other neuronal cells. The role of CCR5Δ32 in PD has not been studied extensively. Huerta and co-workers [90] investigated the influence of various CCR5 polymorphisms in PD susceptibility and progression in a Spanish cohort, using blood as a model. The authors included PD patients as well as late-onset Alzheimer’s disease patients (LOAD) and compared these patient groups to healthy controls. No significant difference in genotypic or allelic frequency distribution between both patient groups and controls was found. The authors thus showed that CCR5∆32 does not contribute to disease susceptibility or clinical outcome for PD and LOAD [90].

Multiple sclerosis is a progressive, immune-mediated disorder that attacks the central nervous system, affecting primarily the brain and spinal cord, causing an impaired flow of information between the brain and the body. The nerves present in the brain and spinal cord are covered with a protective layer, known as the myelin sheath. Demyelination is a process in which the myelin sheath is damaged, resulting in retarded nerve impulses. Lesions, also known as plagues, are an area within the brain which can suffer damage due to inflammation resulting from the immune response in which the myelin sheath is attacked. Ms patients present with demyelination, axonal injury and decreased neurological function [79]. Chemokines and chemokine receptors play a pivotal role in ms by trafficking pro-inflammatory T-cells into the central nervous system. Consequently, mutations within chemokines and receptors may influence disease susceptibility and progression. In a study performed by Gade-Andavolu and co-workers [91], the brain tissue and blood samples of ms patients and healthy controls were obtained. The authors determined the genotypes of the respective samples using PCR. The homozygous and heterozygous CCR5∆32 genotypes were not associated with ms disease susceptibility but were associated with decreased survival rates. In addition, the authors stratified cases by gender and reported a more significant effect of CCR5∆32 on mortality in female patients [91]. Pulkkinen and co-workers [92] obtained blood samples from ms patients and healthy controls and used PCR genotyping data to compare CCR5∆32 distribution among patients and controls. The authors reported increased homozygous CCR5∆32 genotypes within the patient group compared to controls, suggesting this variant to contribute to ms disease susceptibility. Further analysis showed that the increased homozygous CCR5∆32 genotypes were more prevalent in primary progressive ms patients compared to other ms subtypes. In addition, the authors used RT-PCR to determine CCR5 messenger ribonucleic acid (mRNA) levels and flow cytometry to determine the amount of CCR5 expressed on the surface of CD4^+^ T-cells. The authors found that CCR5∆32 reduced the amount of CCR5 expressed on the cell surface and that the amount of CCR5 was not associated with ms subtype or disease progression. Therefore, the authors concluded that the absence of CCR5 on the cell surface is not associated with protection against the development of ms, but that CCR5 is rather involved in the clinical course of ms [92].

Other studies reported that CCR5Δ32 genotypes offer protection against ms due to the involvement of CCR5 in inflammatory responses. Kantor and co-workers [93] performed PCR on blood samples to genotype all individuals in their study and reported that, although CCR5Δ32 does not have an effect on ms disease susceptibility, homozygous and heterozygous CCR5Δ32 genotypes were associated with slower disease progression and progression to disability and therefore a more favourable clinical course. Given the role of CCR5 in inflammatory immune responses and upregulation of CCR5 expression during a diseased state, higher levels of CCR5 may contribute to persistent inflammation which may be damaging [93]. In the study conducted by van Veen and co-workers [94], the authors obtained blood samples of healthy controls and ms patients as well as brain tissue from ms brain donors. Similar CCR5Δ32 distributions among patients and controls were observed. The authors concluded that CCR5Δ32 does not contribute to ms disease development, clinical course, lesion activity, perivascular leukocyte infiltration or axon density. However, the authors found CCR5Δ32 to be associated with a reduced black hole ratio (areas with permanent damage of the axons) and increased remyelination. The authors failed to specify whether these effects are attributed to homozygous or heterozygous CCR5Δ32 genotypes. Therefore, the authors concluded that CCR5Δ32 is associated with a protective phenotype and improved clinical outcome [94].

Some studies found no significant difference in CCR5Δ32 allelic frequency between ms patients and control groups. In these instances, CCR5Δ32 was thus not associated with ms disease susceptibility [95,96,97]. In the investigation performed by Bennetts and co-workers [95], PCR was used to screen an Australian cohort for CCR5Δ32 genotypes. The authors reported similar allelic frequencies of CCR5Δ32 among patients and healthy controls which suggested that CCR5Δ32 is not implicated in ms disease [95]. Kaimen-Maciel et al. [96] obtained blood samples from ms patients who were at various stages of the disease as well as from healthy controls. The authors used PCR to determine CCR5Δ32 genotypes in this Brazilian cohort and reported that ms patients presented with similar CCR5Δ32 frequencies as seen in healthy controls. In addition, CCR5Δ32 frequencies did not differ significantly according to disease stage among ms patients. The homozygous CCR5Δ32 mutation was absent in this cohort. Patients harbouring the heterozygous CCR5Δ32 genotype presented with a later onset of disease. Absence of the mutation in patients was thus protective in this regard. In addition, the authors showed a lack of association of CCR5Δ32 with ms disease susceptibility [96]. Silversides and co-workers [97] obtained blood samples from an Irish population and used PCR to genotype samples and investigate CCR5Δ32 frequencies within the cohort. The cohort included healthy controls and ms patients suffering from various types and stages of disease. The authors found similar genotypic and allelic frequencies of CCR5Δ32 among patients and controls suggesting that CCR5Δ32 is not associated with ms disease susceptibility nor with disease progression. However, when the authors stratified cases according to ms disease types or stages and age of onset, elevated CCR5Δ32 genotypes were observed among patients suffering from relapsing-remitting ms disease. This was especially observed in patients with a known family history of ms disease. This led the authors to report that CCR5Δ32 is associated with an earlier age of onset in relapsing-remitting patients. These results could not be replicated in a group where the family history of remitting-relapsing patients was unknown [97].

### 2.3. Immune System Diseases

The immune system includes organs as well as processes that provide resistance to infection from pathogens. The major organs that form part of the immune system include the thymus, bone marrow and lymph nodes. A dysregulated immune system may cause an overactive or underactive immune response with a pro- or anti-inflammatory fingerprint.

Asthma is considered a chronic inflammatory disease and involves the bronchial tubes within the lungs. Since asthma is a complex condition in which the bronchial tubes are constantly inflamed, chemokines and their receptors are implicated in this disease. The production of chemokines and their receptors is generally upregulated following an allergen challenge. Previous studies reported elevated chemokine levels in patients with asthma [98,99]. Various genetic and environmental factors can also influence the pathogenesis of asthma. The investigation of specific genetic variations and their influence on asthma is thus challenging and contradictory results are often obtained. Hall and co-workers [100] conducted a study regarding CCR5Δ32 and its influence on asthma. The authors obtained a mouthwash sample from Caucasian children and determined CCR5Δ32 genotypes using PCR. Genotypes of randomly selected samples were confirmed by sequencing. The homozygous CCR5Δ32 genotype was absent in children with asthma, whereas the prevalence of asthma was decreased in individuals carrying the heterozygous CCR5Δ32 genotype compared to individuals carrying the homozygous WT genotype. The authors suggested that, based on the prevalence of CCR5Δ32 within their cohort, CCR5Δ32 offers protection. The protective role may be attributed to the development of allergic inflammation [100]. In a later study conducted by Srivastava and co-workers [101], the protective role of the CCR5Δ32 variant was confirmed in childhood asthma, but not adulthood, suggesting that the protective role of CCR5Δ32 might become lost during the transition from childhood into adulthood. Srivastava and co-workers [101] explained that the protective effect might disappear during adulthood due to external factors such as adverse environmental exposures or host factors such as atopy. Atopy describes the genetic predisposition of an individual to develop various allergic reactions and/or disease [102]. This conclusion was made following the analysis of blood samples from a population in northern Scotland. The study firstly genotyped the blood samples of children. Once again, the homozygous CCR5Δ32 genotype was absent in children with asthma. The authors concluded CCR5Δ32 to be associated with protection against asthma in children. The same cohort was used several years later as young adults, but no protective effect was seen [101]. These results were in contrast to the results obtained by Mitchell and co-workers [103] who investigated the association between CCR5Δ32 and asthma in two respective cohorts. The cohorts consisted of individuals from Western Australia and the United Kingdom, respectively. Genotyping of the blood of the respective cohorts showed no significant linkage to asthma in either of the cohorts [103]. Abousaidi and co-workers [104] investigated the frequency of CCR5Δ32 in asthma patients and healthy controls. Blood samples were obtained from an Iranian cohort. Genotypes were determined using PCR. Since asthma is related to an allergic reaction, serum immunoglobulin E (IgE) was measured using an ELISA. The levels of IgE were higher in asthmatic patients compared to controls. While none of the asthmatic patients presented with CCR5Δ32, the heterozygous CCR5Δ32 genotype was present in 1.5% of the control group. Overall, the CCR5Δ32 genotypic distribution among patients and controls was not significant, implying that CCR5Δ32 does not have an influence on the pathogenesis of asthma [104]. In a study by Cracoviensia and co-workers [105] using a Polish population, no statistical difference in allelic frequencies of CCR5Δ32 in asthma patients compared to healthy controls was found. Consequently, the authors identified a lack of association of CCR5Δ32 with asthma [105]. Within the context of asthma and CCR5, the type of immune response induced may influence disease outcome. During allergic inflammation, Th2 cells are mainly involved due to the anti-inflammatory response [105]. Since a larger Th2 response counteracts the Th1 immune response, it may be possible that the function of CCR5, which is primarily expressed on Th1 cells, is suppressed. For that reason, pro-inflammatory chemokine release may be affected which will ultimately result in an altered immune cell signalling pattern and thus contribute to disease pathogenesis. Despite blood as the main sample type analysed, the role of CCR5Δ32 in asthma remains unclear. More studies regarding immune changes based on the genotypes are necessary to further our understanding regarding CCR5Δ32 and its role in asthma disease.

Diabetes mellitus type 2 (T2DM) is also characterised as an inflammatory disease. During this condition, insulin production decreases which leads to insulin resistance as the disease progresses. Muntinghe et al. [106] investigated the effect of CCR5 on cardiovascular mortality in a T2DM patient cohort using blood and urine, respectively. PCR was used to determine the CCR5Δ32 genotypes in the samples of all individuals. Haemoglobin A1c (HbA1c), which shows the ability of the body to control blood sugar, was measured using liquid chromatography. Serum lipid levels as well as serum and urine creatinine levels were routinely tested to determine kidney function and measured using various methods. The authors found that the CCR5Δ32 allele contributed to elevated blood pressure and HDL levels. Surprisingly, patients carrying the CCR5Δ32 allele showed better survival rates. The authors thus concluded that CCR5Δ32 is protective in T2DM. The CCR5Δ32 allele is alleged to protect against other diabetic complications resulting in better survival rates [106]. Since the genotype contributing to the protective effect is not specified, it is suspected that the homozygous genotype would play a bigger role since CCR5 is involved in the initiation of inflammatory responses. Consequently, loss of CCR5 results in less severe inflammatory responses. Our interpretation is supported by the study of Bing et al. [24] who used a rat model to show low levels of CCR5 improved T2DM [24].

Autoimmune diseases refer to a state in which the immune system of an individual produces antibodies against self. There is some evidence indicating that chemokines are involved in autoimmune diseases [107,108]. Genetic variations and mutations in chemokines and their receptors can therefore result in dysregulation of the immune system due to chronic inflammation. Diabetes mellitus type 1 (T1DM) is considered an autoimmune disease. The immune system attacks pancreatic cells responsible for the production of insulin. The inflammatory reaction is supported by the infiltration of CCR5^+^adipose tissue macrophages [109]. Gambelunghe et al. [110] obtained blood samples from an Italian cohort and performed CCR5Δ32 genotyping using PCR and sequencing. The authors reported similar CCR5Δ32 frequencies among diabetic patients and healthy controls and confirmed that CCR5Δ32 was not associated with susceptibility to T1DM [110]. Szalai and co-workers [111] investigated the distribution of CCR5Δ32 among children presenting with T1DM and among nondiabetic individuals. There was no difference in the distribution of CCR5Δ32 among the groups; CCR5Δ32 was therefore not associated with T1DM [111]. Yang et al. [112] used blood from a British cohort and investigated several polymorphisms in the genes of chemokines with a role in T1DM. CCR5Δ32 was not associated with T1DM. However, when combining the effects of various polymorphisms, the authors found that the chemokine system was associated with complications in diabetic patients [112]. Kalev et al. [113] used blood to investigate the frequency of CCR5Δ32 in T1DM and T2DM patients as well as healthy controls using PCR. The authors found no difference in allelic frequencies between patient and control groups. The heterozygous CCR5Δ32 genotype was however associated with the clinical course of T1DM (i.e., there is a later age of onset of T1DM in heterozygous CCR5Δ32 patients compared to homozygous WT patients). With regards to T2DM, heterozygous CCR5Δ32 patients presented with decreased frequency of concomitant diseases compared to homozygous WT patients. The authors finally reported CCR5Δ32 to be associated with protection regarding clinical course, but not disease susceptibility [113]. In a meta-analysis conducted using a mixed population, Song et al. [114] aimed to investigate the role of CCR5Δ32 in diabetes and asthma disease susceptibility. The meta-analysis included fourteen studies of which six were representative of T1DM and three of asthma. In relation to T1DM, CCR5Δ32 was considered a protective factor while no association of CCR5Δ32 with asthma susceptibility was found [114].

Rheumatoid arthritis (RA) is an inflammatory autoimmune disease which causes swelling and deformities in joints. An auto-antibody called rheumatoid factor (RF) is present in the blood of patients. In addition, several immune cells undergo peripheral activation followed by attraction to the synovial compartment. Kohem and co-workers [115] investigated the role of CCR5Δ32 in RA patients and healthy controls as well as the phenotypic expression of CCR5 on T-cells. The authors used flow cytometry to determine the immunophenotype of individual cells. There was no difference in the frequency of CCR5Δ32 among patients and controls. The homozygous CCR5Δ32 genotype was absent in both groups, while the heterozygous CCR5Δ32 genotype was present in some patients. The heterozygous CCR5Δ32 genetic frequencies were similar among patients and controls. The authors found elevated levels of CCR5^+^cells in the synovial fluid of RA patients. Given the role of CCR5 in the immune system, elevated levels of CCR5 enhance inflammation, thus influencing disease severity. The authors observed that heterozygous CCR5Δ32 patients had aggressive RA disease and based on this concluded that CCR5Δ32 was not implicated in RA disease [115]. Lindner and co-workers [116] investigated the association of CCR5Δ32 with RA and juvenile idiopathic arthritis (JIA). The authors reported no association of CCR5Δ32 with both diseases based on similar CCR5Δ32 genetic frequencies among patients and controls [116]. Some studies report that CCR5Δ32 results in less severe disease and improved clinical outcome in RA patients thus offering a protective role. In a study conducted by Garred and co-workers [117] patients carrying either the homozygous or heterozygous CCR5Δ32 genotypes tested negative for RF compared to patients with the homozygous WT genotype. In addition, the prevalence of swollen joints and morning stiffness was decreased in these patients and suggests CCR5Δ32 may influence autoimmune disease severity by improving clinical course [117]. Gómez-Reino et al. [118] used blood samples from RA patients, systemic lupus erythematosus (SLE) patients and healthy controls. The authors performed PCR to determine CCR5Δ32 genotypes. The authors reported that none of the RA patients presented with the homozygous CCR5Δ32 genotype, while the heterozygous CCR5Δ32 genotype and homozygous WT genotype caused the same severity in disease. The results obtained suggest a protective role of CCR5Δ32 against RA. In addition, the authors investigated the role of CCR5Δ32 in SLE; similar CCR5Δ32 genotypic and allelic frequencies were observed among patients and controls suggesting a lack of association of CCR5Δ32 with SLE [118]. In a study based on a New Zealand cohort, Pokorny and co-workers [119] performed PCR on blood samples to ultimately compare the frequencies of CCR5Δ32 between Caucasian RA patients and healthy controls. None of the RA patients presented with the homozygous CCR5Δ32 genotype. The frequency of the heterozygous CCR5Δ32 genotype was less in RA patients compared to controls. This evidence suggests a protective role of the homozygous CCR5Δ32 genotype. Although the allelic frequency of CCR5Δ32 was reported to be decreased in patients, the authors reported that frequency of CCR5Δ32 did not differ significantly when cases were stratified according to disease severity or outcome [119]. Rossol et al. [120] reported decreased RA disease susceptibility for German individuals carrying CCR5Δ32. For this, the authors determined the prevalence of CCR5Δ32 in RA patients as well as healthy controls. Blood samples were obtained and genotypes were determined using PCR. The authors found that CCR5Δ32 was more prevalent in RA patients with articular, inside joints diseases when compared to RA patients with extra-articular, outside joints manifestations. The authors also used immunofluorescence and immunohistochemistry to determine levels of various antibodies and nephelometry to determine levels of RF in serum. The authors found that CCR5Δ32 was associated with lower RF levels. In conclusion, it was shown that CCR5Δ32 offers protection against joint erosions and extra-articular manifestations. The authors thus demonstrated that RA disease susceptibility as well as clinical course is influenced by an individual’s CCR5Δ32 genotype [120].

Systemic lupus erythematosus is a chronic autoimmune disease more commonly known as lupus. Multiple organs are affected in this disease with inflammation mainly attributed to chemokines. Lupus patients present with elevated chemokine levels during active disease [121]. The role of CCR5∆32 in the development of SLE was investigated in a study conducted by Carvalho et al. [122] using a Portuguese cohort. Blood samples were obtained and subjected to PCR analysis to determine sample genotypes. The authors showed that the frequency of heterozygous CCR5∆32 for SLE patients was lower compared to the control group, while the homozygous CCR5∆32 genotype was absent among patients. Since the mutation did not feature much in patients, the authors reported CCR5∆32 to be associated with protection against SLE disease [122]. Schauren et al. [123] investigated the association of CCR5∆32 with SLE in a Brazilian cohort. The CCR5∆32 genotypic frequencies were decreased in European patients and increased in African patients, respectively compared to healthy controls. The homozygous CCR5∆32 genotype was absent in European patients. The authors therefore showed that CCR5∆32 genotypes are involved in the protection of European patients against SLE. In addition, the authors showed that CCR5∆32 genotypes are associated with development of class IV lupus nephritis [123]. Similar results were obtained by Baltus and co-workers [124] in a female Brazilian cohort. The authors investigated the association of CCR5∆32 genotypes with SLE disease susceptibility and disease activity. Blood samples were obtained and genotypes were determined using PCR. The authors reported increased homozygous and heterozygous CCR5∆32 frequencies within SLE patients suggesting that CCR5∆32 is associated with the development of SLE disease. Based on clinical information obtained from all individuals, homozygous and heterozygous CCR5∆32 were found to be associated with earlier age of onset. Disease-associated markers were measured using immune-based methods. When the authors adjusted the results for the ethnicity of the patients, the authors showed that ethnicity influenced autoimmune responses. However, CCR5∆32 was not associated with disease activity [124]. Lupus nephritis (LN) refers to kidney inflammation and is often a result of SLE. The role of CCR5 in kidney inflammation is established (i.e., CCR5^+^T-cells involved in immune responses cause scarring or thickening of interstitial tissues known as fibrosis) [125]. A meta-analysis conducted by Zhou and co-workers [126] revealed no association of CCR5∆32 with LN susceptibility in Asians and Caucasians. This result is based on five studies included in the meta-analysis. An increased risk of developing LN disease in African patients was reported in one study included in the meta-analysis. Overall, Zhou and co-workers [126] found no difference in CCR5∆32 gene distribution among SLE patients and LN patients, suggesting that CCR5∆32 cannot predict whether SLE patients will develop LN.

### 2.4. Infectious Diseases

Infectious diseases are caused, amongst others, by viral and parasitic organisms. Clearance of the pathogen is dependent on an effective immune response. Given the role of chemokines and chemokine receptors in the immune response, CCR5 is implicated in many infectious diseases.

#### 2.4.1. Acquired Immunodeficiency Syndrome

In 1995 a paper was published describing the role of CCR5 in HIV-1 infection. Given their competitive binding to the CCR5 receptor, the chemokines MIP-1α, MIP-1β and CCL5 (RANTES) were shown to inhibit HIV-1 infection in cells [127].

The presence of a virus is generally detected by measuring the antibodies present in the blood against that specific virus. In the case of HIV-1, the virus can be present in the blood of individuals carrying the homozygous CCR5Δ32 genotype resulting in a seropositive test. However, given the role of CCR5 as a coreceptor for HIV-1 to enter the cell, decreased levels of CCR5 on the cell’s surface limit viral entry. Therefore, seropositive individuals with the homozygous CCR5Δ32 genotype are still considered protected. In a meta-analysis conducted by Dean and co-workers [30], the homozygous CCR5∆32 genotype was present exclusively in a group of HIV-exposed, but uninfected individuals. The authors reported increased frequencies of the heterozygous CCR5∆32 genotypes in patients that survived HIV-1 infection for more than ten years. Survival analysis showed slower disease progression in heterozygous CCR5∆32 patients. Therefore, homozygous CCR5∆32 was associated with protection against HIV-1 infection, whereas the heterozygous CCR5∆32 genotype was associated with slower HIV-1 disease progression [30]. Samson et al. [31] showed the CCR5∆32 genotype to occur more frequently in Caucasian populations while being absent in African and Japanese populations. The homozygous CCR5∆32 genotype was absent in Caucasian patients, while the frequency of heterozygous CCR5∆32 genotypes was lower in HIV-infected patients. One homozygous CCR5∆32 carrier donated white blood cells and the authors showed that HIV-1 entry was inhibited which confirmed CCR5 to be the main coreceptor for HIV-1. The authors concluded that the decreased frequencies of heterozygous CCR5∆32 in seropositive patients suggest partial resistance against HIV-1 infection thus affording protection [31]. In a South African-based study done by Jaumdally et al. [33], the immune profiles of samples carrying protective and non-protective haplotypes were investigated. The authors investigated the interaction between systemic inflammation, immune activation of T-cells and CCR5 genotype in context to HIV-1 resistance. PCR, immune assays and flow cytometry were applied to measure genetic and immune parameters. The authors showed CCR5Δ32 frequency to be similar among patients and controls. Patients bearing the protective haplotype presented with decreased levels of immune activation, CCR5 expression and plasma cytokines [33]. Marmor and co-workers [37] investigated HIV resistance in exposed but uninfected and infected cohorts, respectively. The cohort consisted of patients carrying either the homozygous or heterozygous CCR5∆32 genotype. The individuals carrying the homozygous CCR5∆32 genotype were exposed but not infected, while the heterozygous CCR5∆32 group was infected and showed slow disease progression. The authors documented both genotypes to be associated with protection against HIV-1 infection [37]. Liu and co-authors [128] isolated CD4^+^ T-cells from two individuals that were exposed to HIV, but who remained uninfected. The authors showed that these cells remained resistant to HIV infection, despite being infected with T-cell line-adapted viruses. Subsequent analysis revealed both patients’ cells to carry the homozygous CCR5∆32 mutation. The authors concluded CCR5 to be implicated in HIV infection and disease progression, more specifically, that homozygous CCR5∆32 genotypes confer resistance to HIV infection [128]. Rana and co-workers [129] used human primary blood cells to show that the homozygous CCR5∆32 mutation results in the failed expression of functional CCR5 protein on the cell surface and that CCR5 is essential for HIV entry into the cell [129].

Balotta et al. [130] obtained blood samples from a cohort consisting of HIV-infected and uninfected individuals. PCR was used to determine CCR5∆32 genotypes. Surprisingly, the authors observed one homozygous CCR5∆32 genotype among HIV-infected patients which was confirmed through sequencing. In this instance the homozygous CCR5∆32 genotype was found to not confer absolute protection against HIV infection [130]. Several independent studies conducted on the blood samples of males confirmed that the CCR5∆32 homozygous genotype provides incomplete protection against HIV infection [129,130,131,132,133,134,135]. In all these studies genotyping was done using PCR and/or sequencing. In all of the samples HIV infection was also confirmed whether through Western blotting techniques [131], immunoassays (e.g., p24 assay), or measuring viral load [132], to name a few. Non-protection was ascribed to the fact that patients were infected with a dual-tropic virus, using receptors other than CCR5 [130,131,132,133,134,135,136]. A mutation of the stromal cell-derived factor 1 (SDF-1) gene, which generally serves to protect against HIV-infection, was also documented [136]. Collectively, these studies showed that CCR5Δ32 offers protection only in the case of macrophage-tropic HIV-1 infection. T-cell tropic HIV primarily uses the CXCR4 receptor, whereas dual-tropic viruses can use both CXCR4 and CCR5. Where patients are infected with T-cell and/or dual-tropic viruses as in the case of several studies reported here [129,130,131,132,133,134,135], CCR5Δ32 offers no protection. In fact, risk for infection is increased.

With respect to HIV-1 infection, the protective role of CCR5Δ32 in context to macrophage-tropic HIV infection, which makes use of CCR5 to gain entry into the cell, is thus clear and well established.

#### 2.4.2. West Nile Fever

The West Nile virus (WNV) is a single stranded RNA virus and is considered the causative agent of West Nile fever (WNF). In general, WNV infection is asymptomatic while severe cases can result in meningitis or encephalitis. A study conducted by Glass et al. [137] showed that mice with a homozygous WT CCR5^+/+^ genotype, which is similar to the CCR5 WT genotype in humans, had a better survival rate than CCR5-deficient mice. In these mice there was increased expression of CCR5 which is required for lymphocyte recruitment into the central nervous system [137]. The same group conducted a study in 2006 using the blood and cerebrospinal fluid of Caucasians in which they established that the CCR5∆32 homozygous genotype (generally associated with low CCR5 levels) is a risk factor for symptomatic WNV infection (minimal CCR5 for lymphocyte recruitment) [138]. Similar conclusions were made in the meta-analysis of Lim and co-workers in 2008 [139]. The findings for this disease are thus consistent, independent of the model used.

#### 2.4.3. Hepatitis B Infection

Hepatitis B virus (HBV) infection is the causative agent of severe liver infection which may be fatal. Chronic HBV infection can progress and result in cirrhosis as well as hepatocarcinoma. Activation of Th1 lymphocytes is necessary during viral infections. The main immune cells involved in eliminating viral infections are the CD8^+^ T-cells [140]. Chemokines play a pivotal role in leukocyte migration and viral eradication. Nevertheless, constant expression of certain chemokines affects immune activation. Persistent immune activation may in turn result in tissue damage while varying levels of CCR5 expression can influence immune responses [141,142]. Immune cells migrate to the HBV-infected liver due to the presence of CCR5 receptors on the surface of cells [143]. In a study performed by TrehanPati and co-workers [144], blood samples were obtained from healthy controls, acute HBV infection and chronic HBV infection patients. The authors did not mention the ethnic group(s) used for their study. RT-PCR was used to analyse CCR5 expression profiles while flow cytometry was used to type CD4^+^ T-cells. Various antibodies related to HBV disease were detected using ELISA. The authors showed that T regulatory cells were more abundant during the acute phase of HBV disease when compared to chronic HBV patients and healthy controls. In addition, the authors showed increased CCR5 expression levels on CD4^+^ cells during acute HBV infection which might be implicated in viral clearance and offering protection against disease. [144]. A group of researchers investigated CCR5 expression on the T lymphocytes of Iranians in two respective studies. In the first study conducted by Arababadi et al. [145], the rate of CCR5 expression on natural killer (NK) cells was determined. The authors tested for various antibodies related to HBV infection and flow cytometry analysis was performed to evaluate CCR5 levels. Low levels of CCR5 on NK cells of HBV patients were associated with reduced viral clearance signifying non-protection against the disease [145]. Arababadi et al. [146] investigated the expression of CCR5 on CD8^+^ T-cells in HBV patients. The CCR5Δ32 mutation was not detected in any patients. The authors found reduced CCR5 levels on CD8^+^ T-cells, unrelated to the CCR5Δ32 mutation, suggesting that HBV has an influence on CCR5 expression. Therefore, the authors concluded that viral and host genetics can impact CCR5 expression resulting in reduced viral clearance [146]. Ahmadabadi et al. [147] (not summarised in Table 1) investigated CCR5 expression levels on CD8^+^ T-cells in chronic HBV patients. Various immune markers related to liver function were measured using ELISA-based assays. CCR5 levels were determined using flow cytometry analysis. The authors reported a reduced percentage of CD8^+^ T-cells in the chronic HBV patients. Surprisingly, the amount of CCR5 on CCR5^+^/CD8^+^ T-cells was increased in the chronic HBV group. The chronic HBV group also presented reduced levels of total lymphocytes. Similar to the findings of Arababadi [145], the lower frequency of CCR5^+^/CD8^+^ cells in chronic HBV patients was found insufficient to eradicate HBV from the hepatocytes and was therefore characterised as non-protective [147]. These results however contrast with an earlier study performed by Thio and co-workers [148] which showed that the homozygous CCR5∆32 polymorphism offers genetic protection for patients recovering from HBV infection. Eight out of nine CCR5Δ32 homozygotes in this cohort recovered from infection. In addition, CCR5∆32 was frequent in the recovery group and could thus be associated with protection regarding clinical outcome [148].

Ellwanger and co-workers [149] investigated the role of CCR5Δ32 in HBV mono-infection and HBV/HIV-1 co-infection. Blood was obtained from a Brazilian cohort. Genotypic analysis was performed using PCR. Patients were grouped according to HBV, HIV-1 and HBV/HIV-1 infection and CCR5Δ32 allelic frequencies were compared to that of healthy controls. Similar genotypic frequencies were observed among groups. The authors found that the CCR5Δ32 allele was not associated with HBV infection susceptibility, but that it did offer some protection against HBV/HIV-1 co-infection. The authors noted that the protective effect observed might be due to the known protective effect of CCR5Δ32 against HIV-1 infection [149].

#### 2.4.4. Hepatitis C Infection

Hepatitis C virus (HCV) infection leads to inflammation of the liver, which ultimately results in chronic liver disease as well as liver damage. It is established that chemokines play a pivotal role in chronic HCV infection. More specifically, CCR5 is involved in promoting cellular responses such as inflammation in the liver and during fibrogenesis. During the acute phase of infection, chemokines are involved in chemoattraction of effector T-cells to the site of infection in the liver in order to aid viral clearance. During the chronic phase of infection, the inflammatory cells present at the site of infection aggravate liver cell injury which ultimately results in the progression of chronic HCV to fibrosis. CCL5 is involved in the regulation of the migration of immune cells and also serves as a ligand for several other C-C chemokine receptors besides CCR5. In an *in vitro* study, CCL5 expression was upregulated by the presence of full-length HCV [150] or the viral envelope protein [151]. The secreted chemokines required interaction with CCR5 in order to exert an inflammatory response. Studies have however found that the interaction between CCR5 and CCL5 may result in reduced expression of CCR5 on the cell surface as a result of receptor internalization, an endocytotic process in which the receptors are relocated from the cell surface to the inside of the cell [151,152]. Lichterfeld and co-workers [153] obtained blood samples from chronically-infected HCV patients and healthy controls, respectively. The authors sought to determine whether CCR5 levels may be affected by HCV disease instead of CCR5Δ32 genotypes. The authors confirmed CCR5Δ32 genotypes using PCR and determined CCR5 expression on T-cells using flow cytometry. Chemokine levels were measured using ELISA. In addition, the levels of mRNA for the CCR5 protein were detected using RT-PCR. All patients carried a homozygous WT CCR5 genotype. The authors showed that the CD8 T-cells of healthy controls presented with increased levels of CCR5 compared to HCV patients. Levels of CCR5 mRNA were not altered and therefore did not influence CCR5 expression on the cell surface. Due to the absence of CCR5Δ32 genotypes in patient samples, the authors suggest that receptor internalization best explains the reduced amount of peripheral CCR5^+^ cells in chronic HCV patients. [153]. Hellier et al. [154] sampled liver biopsies from a group of patients to investigate several CCR5-related polymorphisms and their association with persistent HCV carriage, liver disease severity, hepatic inflammation and response to treatment. The authors confirmed that patients were free of liver disease that derived from HCV infection. The authors reported that, among patients, homozygous CCR5Δ32 genotypes were found to be associated with reduced portal inflammation and less severe fibrosis, and suggested CCR5Δ32 to be associated with HCV infection clinical outcomes [154]. Promrat and co-authors [155] investigated several polymorphisms linked to the chemokine system using the blood of liver disease patients and healthy controls, respectively. Most liver disease patients suffered from HCV infection. The authors reported the frequencies of homozygous CCR5Δ32 genotypes to be similar among patients and controls and therefore not associated with HCV disease susceptibility. Furthermore, CCR5Δ32 was not associated with clinical parameters related to HCV infection [155]. Wald et al. [156] investigated the role of CCR5Δ32 genotypes and inflammation during the early stages of HCV disease. Blood and liver biopsies were obtained from all study participants. In addition, inflammatory activity was assessed by a hematopathologist during liver biopsy collection. CCR5Δ32 genotypes were determined using PCR. The authors identified the heterozygous CCR5Δ32 and homozygous WT genotypes. Based on similar frequencies of CCR5Δ32 genotypes among patients and healthy controls, the authors reported that CCR5Δ32 was not associated with HCV infection susceptibility. Based on inflammatory activity scores, CCR5Δ32 genotypes do not influence disease progression towards end-stage liver disease. However, when the authors stratified patients according to CCR5 genotypes, the heterozygous CCR5Δ32 genotypes were associated with reduced inflammation during early stages of HCV disease [156]. Ruiz-Ferrer et al. [157] used blood and liver biopsies of a Spanish population to investigate the involvement of chemokine system-related polymorphisms in the response to HCV infection. PCR was used to determine CCR5Δ32 genotypes. None of the patients carried the homozygous CCR5Δ32 genotype. The authors found no significant difference in the CCR5∆32 allelic frequencies of patients when compared with controls, hence no association of CCR5Δ32 to HCV infection, liver damage or clinical outcome [157]. Goulding and co-workers [158] investigated the role of CCR5∆32 to HCV in a British population. The authors obtained blood samples for genotyping and liver biopsies to determine histological changes. Genotyping was performed using PCR, while HCV antibody status was analysed using an ELISA. The authors compared the frequencies of several chemokine and chemokine receptor polymorphisms with disease severity in HCV patients and healthy controls. The frequency of the heterozygous CCR5Δ32 genotype in both HCV patients and controls was similar [158]. The heterozygous CCR5Δ32 genotype was associated with spontaneous elimination of HCV. In addition, individuals with the heterozygous genotype presented with decreased hepatic inflammation. Reduced inflammation and elimination of HCV suggested protection against HCV disease.

#### 2.4.5. Chagas Disease

Chagas disease is an inflammatory infectious disease caused by the parasite *Trypanosoma cruzi.* Transmission occurs mainly via insects. The parasite is disseminated by the immune system and, in some cases, ultimately results in the development of potentially fatal CM [159]. The role of chemokines and chemokine receptors has been studied in Chagas disease in several populations. A recent study demonstrated several genes involved in the immune response to influence the severity of Chagas disease [160]. The role of chemokines in the early stages of Chagas-induced CM is well established. In a study conducted by Talvani et al. [161], blood samples were obtained from non-infected individuals, patients suffering from mild Chagas-induced CM and patients suffering from severe Chagas-induced CM. Flow cytometry was used to determine the levels of CCR5 on peripheral blood mononuclear cells. Patients suffering from mild Chagas-induced CM presented with elevated CCR5 expression on leukocytes compared to patients suffering from severe Chagas-induced CM and non-infected individuals. In addition, the amount of CCR5 correlated with disease severity (i.e., reduced CCR5 levels were found in patients suffering from severe Chagas-induced CM which related to decreased heart function). The authors concluded that CCR5 is involved during the early stages of Chagas-induced CM, highlighting the role of the chemokine system in Chagas-induced CM [161]. Fernandez-Mestre et al. [162] obtained blood from a Venezuelan cohort and determined CCR5Δ32 genotypes using PCR. The authors confirmed *T. cruzi* infection with immunofluorescence. The homozygous CCR5Δ32 genotype was absent. Allelic and genotypic frequencies were similar among asymptomatic and symptomatic patients. It was concluded that CCR5Δ32 was not associated with Chagas disease. However, further studies are required due to the low prevalence of CCR5Δ32 in Venezuelan individuals [162]. Due to the low prevalence of CCR5Δ32 within selected cohorts, other studies found polymorphisms (other than CCR5Δ32) within the CCR5 gene to influence Chagas disease susceptibility and progression [159,163,164,165]. Collectively, these results indicate that further studies are required to elucidate the role of CCR5 in the presence of Chagas disease.

#### 2.4.6. Influenza A

Influenza, an acute viral respiratory infection, promotes a severe pro-inflammatory cytokine and chemokine response in lung tissue. Influenza A is the most common type of influenza, also known as “the flu”. It is caused by the influenza virus for which there are several strains. Following infection, an inflammatory response is initiated in order to limit viral replication. Pro-inflammatory mediators form the majority of the immune molecules present at the site of infection [166]. The inflammatory response results in recruitment of antigen-specific memory CD8^+^ T-cells and is essential to regulate infection and viral replication [167]. However, instead of offering protection, a variety of cell types can have a negative influence on patient outcome when present in large quantities [168]. Disease outcome can thus vary based on differential inflammatory response patterns. In a study conducted by Kohlmeier and co-workers [167] (not summarised in Table 1), the authors used a mouse model to investigate the role of certain chemokine receptors in the recruitment of virus-specific memory CD8^+^ cells. In addition, the influence of chemokine-receptor deficiency on an effective cellular response was also investigated. The results suggested that CD8^+^ T-cell recruitment depends on CCR5 expression. CCR5 is therefore crucial for an effective immune response [167]. Swine flu is a respiratory disease caused by a specific strain of the influenza virus known as H1N1. Keynan and co-workers [169] investigated the role of CCR5Δ32 genotypes in H1N1-infected patients. Blood was obtained from patients comprising various ethnicities. In patients suffering from severe disease, an increase in the prevalence of heterozygous CCR5Δ32 genotypes was observed. The heterozygous CCR5Δ32 genotypes were associated with disease severity, but not disease susceptibility [169]. In a study conducted by Sironi et al. [170], blood was obtained from several H1N1-infected patients presenting with various clinical symptoms. The CCR5Δ32 genotypes were determined using PCR. One individual presented with the heterozygous CCR5Δ32 genotype and mild disease. CCR5Δ32 therefore did not influence influenza A disease severity in a significant way [170]. In an investigation by Maestri and co-workers [171] which comprised a Brazilian cohort, no significant difference was found in either allele or genetic frequencies of CCR5Δ32 in hospitalised patients compared to non-hospitalised patients. The homozygous and heterozygous CCR5Δ32 genotypes were thus not associated with influenza A (H1N1) [171]. Similar results were obtained by Matos et al. [172] who used the nasopharyngeal swabs and post mortem specimens of a Brazilian cohort (i.e., no significant difference in the distribution of the homozygous WT and heterozygous genotypes as well as CCR5Δ32 allelic frequencies in the different clinical groups were found). The findings highlight a lack of association of CCR5Δ32 with disease susceptibility, severity or mortality rates in a Brazilian cohort [172].

The aforementioned studies highlight the contrasting role(s) of CCR5Δ32 with regards to diseases. While several studies concluded the CCR5Δ32 genotype to be associated with increased susceptibility to disease due to impaired immune signalling, thus preventing effective viral clearance, further studies are required to clarify this.

### 2.5. Cancer

Cancer is a broad term used to describe a diseased state in which cells show an abnormal growth pattern. Certain changes result in immortal cells that undergo uncontrolled growth and division. There are various cancer types with some more severe than others. The type of cancer is characterised by the localization of the immortal cells. The tumour environment contains important elements such as chemokines and chemokine receptors [173]. The chemokines produced and secreted by cancer cells play a pivotal role in leukocyte infiltration [174]. In a study conducted by Lavergne and co-workers [175], the authors used a mouse model to determine whether CCL5 can control the growth of tumours. CCL5 expression was induced in these mice. In CCR5-deficient mice, decreased CCL5 levels were observed and anti-tumour effects were absent. The results of this study therefore support the idea that CCL5 together with CCR5 plays an important role in the recruitment of immune cells to tumour sites and ultimately delays tumour growth [175]. Systems lacking the CCR5-CCL5 interaction are thus at risk of cancer development and tumour growth. Gao and co-workers [22] used a mouse model to demonstrate the enhanced proliferation of breast cancer cells upon CCL5-CCR5 activation. Cell lines were used to show that the presence of CCL5 activates CCR5 and ultimately results in increased glucose uptake and ATP production. Ultimately, these metabolic changes resulted in increased proliferation of cancer cells [22]. In a later study, Gao et al. [176] again showed that CCL5-CCR5 interactions influence metabolic events during tumour onset which ultimately enhance the formation of new tumours, a process known as tumorigenesis. This conclusion was made after the authors showed that, in the absence of CCR5, tumour progression is delayed. The authors made use of advanced techniques, such as chromatography and mass spectrometry, to measure the altered metabolism [176].

The role of CCR5∆32 in breast cancer in an Iranian cohort was investigated by Eskandari-Nasab and co-workers [177]. Blood samples were collected and genotyped. Homozygous and heterozygous CCR5∆32 were not associated with breast cancer development [177]. A meta-analysis conducted by Li and co-workers [178] included six breast cancer studies. Three of these studies used Caucasian cohorts. Two studies used Asian cohorts, and one study used a Brazilian cohort. The authors reported CCR5∆32 to not be associated with susceptibility to breast cancer. This finding was consistent even when the authors stratified cases according to ethnicity. The authors failed to specify whether CCR5∆32 was present as homozygous or heterozygous genotypes. [178]. More recently, a study conducted by Jiao et al. [179] reported on a possible protective role of CCR5 in breast cancer as investigated in a mouse model. Flow cytometry was used to sort cells according to CCR5 expression. These cells were then implanted in mice; some mice received CCR5^+^ cells, whereas other mice received CCR5-negative (CCR5^-^) cells. CCR5 is involved in various cell signalling pathways. The regulation of pathways involved in DNA repair is altered in CCR5^+^ and CCR5^-^ cells. The authors showed mice harbouring CCR5^+^ cells to have an improved activity of pathways involved in DNA repair. Consequently, the authors concluded that DNA damage repair can be induced by the presence of functional CCR5 due to the cell signalling pathways affected [179]. In this context, the CCR5^+^ cells serve a protective role.

Degerli and co-workers [180] conducted an investigation regarding the role of the heterozygous CCR5∆32 genotype in various carcinomas in a Turkish population. Carcinomas typically originate from tissues lining the body’s outer and inner surfaces. In this study, the various carcinomas studied included: breast, laryngeal, thyroid and brain. The study also included healthy individuals as controls. The heterozygous CCR5∆32 genotype was more prevalent in breast cancer patients, but it was not statistically significant when compared to the prevalence of CCR5Δ32 in other carcinomas studied. The authors concluded that CCR5∆32 was not associated with carcinomas, but may be a risk factor for the development of breast cancer [180]. Duell and co-workers [181] investigated the role of various mutations in pro-inflammatory genes as well as the role of CCR5 in the development of pancreatic cancer. With regards to CCR5, no association was found between CCR5∆32 and pancreatic cancer risk. However, the authors did report a possible interaction between CCR5∆32 and active smoking. The data obtained suggested pancreatic cells to be partially protected against the effects of tobacco smoke by intact CCR5. Mutations such as the 32 bp deletion within the CCR5 gene generally result in non-functional receptors, reduce the secretion of tobacco-induced inflammatory mediators and can therefore be considered as a risk factor for pancreatic cancer [181].

Singh and co-authors [182] investigated the role of CCR5∆32 in the development of cervical cancer. The authors used cervical carcinoma biopsies and found a non-significant higher frequency of heterozygous CCR5∆32 in patients when compared to controls. Homozygous CCR5∆32 genotypes were absent among patients. CCR5∆32 was found to not be a risk factor for the overall development of cervical cancer. Nevertheless, the risk for early stage cervical cancer may be influenced by this mutation, but further studies are necessary [182]. In a study conducted by Zheng and co-workers [183], the possible association of CCR5∆32 with cervical cancer and human papillomavirus (HPV) was investigated. Cancer induces an immune response which is exacerbated by the presence of HPV. The overactive and long-term immune response may influence cells to become cancerous due to constant cell signalling and the inflammatory mediators secreted. In the Swedish cohort studied, the frequency of CCR5∆32 was increased among HPV^+^ cervical cancer patients compared to HPV^-^ cervical cancer patients. CCR5∆32 may therefore be a risk factor for HPV infection and thus increase the risk for cervical cancer [183].

Srivastava and co-authors [184] investigated the influence of CCR5∆32 on the development of gallbladder cancer. Blood samples were used to investigate the CCR5 genotype distribution in patients and healthy controls. The heterozygous CCR5∆32 genotype was more frequent in patients compared to controls. When the authors stratified the cases according to the presence of gallstones, they showed that both homozygous and heterozygous CCR5∆32 genotypes are associated with a risk for gallbladder cancer irrespective of the presence of gallstones. CCR5∆32 did not have an influence on disease susceptibility based on gender, but may be implicated in the early development of gallbladder cancer [184].

The role of CCL5 in the progression of gastric cancer was investigated by Sugasawa and co-workers [185]. The data revealed that gastric cancer cells may have an influence on the level of CCL5 secretion by CD4^+^ T-cells. Cancer cells express receptors for CCL5 and increased levels of CCL5 are present in cancerous tissue. The increased expression of CCL5 activates CCR5 and ultimately results in tumour progression by means of increased cancer cell proliferation, while the elimination of tumours by CD8^+^ T-cells is inhibited. The authors thus showed that CCR5 expression is associated with a worse prognosis [185]. A study regarding mutations in chemokine and chemokine receptor genes and their influence on gastric cancer development and survival was conducted by Gawron and co-workers [186]. Blood samples were used from a cohort consisting of individuals from Poland. The authors found no association between CCR5∆32 and gastric cancer risk or survival outcome [186]. In contrast, a study conducted by Cao and co-workers [187] revealed that CCR5 may contribute to gastric cancer metastasis, a process in which some cancer cells separate from the tumour to form secondary tumours at a different location. The authors showed that metastatic gastric cancer tissues present with increased CCR5 levels compared to gastric cancer without metastasis. Immunohistochemical staining was used to determine the levels of chemokines and chemokine receptors. Th1 and Th2 cytokines were measured using RT-PCR and the authors concluded that CCR5 may influence the Th1/Th2 balance [187].

The role of various chemokines and chemokine receptors in bladder cancer was investigated by Kucukgergin and co-workers [188] using a Turkish population. Bladder tumour biopsies were used. The authors reported an association of the heterozygous CCR5∆32 genotype with increased risk of bladder cancer development [188].

In another investigation conducted by Kucukgergin and co-workers [189], the role of CCR5∆32 in prostate cancer was investigated. Blood samples from a male Turkish population were collected and PCR-RFLP was used to determine sample genotypes. The cohort did not contain individuals carrying a homozygous CCR5∆32 genotype. The authors reported that the heterozygous CCR5∆32 genotype was associated with an increased risk of developing prostate cancer [189]. In contrast, Zambra and co-workers [190] investigated the role of CCR5∆32 in a Brazilian population using DNA samples derived from blood. The authors reported no association of either homozygous or heterozygous CCR5∆32 genotype with prostate cancer development, nor with the clinicopathologic status [190]. Similar results were obtained in an earlier case-control study by Petersen and co-workers [191] in a larger Australian cohort. Blood samples were obtained from participants and subjected to PCR analysis to determine CCR5∆32 genotypes. Similar allelic frequencies were observed among patients and healthy controls. The authors thus concluded CCR5∆32 to not be associated with susceptibility to prostate cancer [191].

Acute myeloid leukemia (AML) is a type of cancer which affects the bone marrow and normal formation of various blood cells. Khorramdelazad and co-workers [192] investigated the prevalence of CCR5∆32 within Iranian patients suffering from AML. Neither the homozygous nor heterozygous CCR5∆32 genotypes were present in the patient group, while three individuals in the control group presented with the heterozygous CCR5∆32 genotype. The authors concluded a lack of association of CCR5∆32 with AML susceptibility in an Iranian cohort [192]. To date, the role of CCR5 in various cancers remains unclear.

## 3. Conclusions

In this article, the main focus was to review the role of CCR5 particularly in disease since genotype influences the expression and/or the amount of CCR5 present on the cell surface, susceptibility to infection and disease pathogenesis. Taken together, CCR5 is crucial for mediating signal transduction and activating immune responses which ultimately impact the cell’s metabolism. Upon reviewing the role of CCR5, frequency of the receptor, genotype and/or levels of CCR5 were frequent terminologies encountered in the literature. While the CCR5∆32 genotype is synonymous with CCR5 levels, this relationship does not hold for allelic frequency. While the role of CCR5 in some diseases such as HIV-1/AIDS is clearer, it is contradictory in others. The different findings may be attributed to various factors such as differences in study design (experimental vs. meta-analysis) as well as sample size. Most often, meta-analysis yielded no association between CCR5∆32 and disease. Given that the information from varied studies are “pooled” as part of a meta-analysis, some of the associations that may exist in individual experiments are possibly overshadowed when reported in this way.

In the case of cardiovascular diseases, central nervous system diseases and cancer for example, the sample matrix and/or model depicts different roles for CCR5. Blood is often the choice of sample since it is acquired easily using minimally invasive techniques. Furthermore, genomic DNA can easily be extracted for genetic analysis. However, while blood was the more common matrix analysed (Table 1), it mostly showed no association to CCR5 while a localised sample such as brain tissue often defined CCR5’s protective vs. non-protective roles better. Similarly, protective and non-protective roles were often better characterised in animal (mice) models. Contrasting roles for the receptor were also evident when the samples of different ethnic groups and/or from different geographical regions as well as different genders or age were compared. The type of immune response, number of cells, type of immune cells and biological processes such as internalization all have an impact on CCR5 levels and function and may ultimately influence disease pathogenesis and outcome. Depending on whether an adult or paediatric model was used, the role of CCR5 in the disease was different. With respect to cancer, CCR5 is associated with different outcomes depending on the type of cancer and/or tumour investigated and its associated microenvironment.

From Table 1 it is also evident that most of the assays used to measure CCR5 status and/or its effects comprised of molecular-type assays. The PCR and RFLP methods were the most common methods used. Both methods are easy to do, time-efficient and require low sample volumes relative to sequencing. Immune analysis was limited as was the recording of metabolic parameters. Where immune and metabolic data were recorded, investigators mostly reported on select molecules using older conventional assays.

Variable phenotypic presentation is common in most diseased states (i.e., patients with the same disease often present with different symptoms). This review and the research of others [40,53] highlights the role of CCR5 in various diseases. The increased biological characterisation of CCR5 may however clarify the contrasting roles of this molecule. Gaining knowledge regarding the altered immune and metabolic profiles due to differential CCR5 status of uninfected and infected models will ultimately improve our understanding of the receptor.

Metabolomics is defined as the study of metabolites [193] and offers the ability to link CCR5 homozygous WT, homozygous mutated and/or heterozygous genotypes with phenotype (e.g., CCR5 levels, frequency, etc.). In living organisms, small molecules known as metabolites are continuously chemically transformed in various metabolic pathways. Measuring metabolite concentrations can thus inform on the biochemical activity of cells and its associated phenotype(s) [194]. Using a metabolomics approach, metabolites can be detected in samples displaying differential CCR5 status. These metabolites can be identified and quantified in an unbiased manner in several different biofluids. Indeed, CCR5 has been shown in a CCR5-deficient mouse model during *Toxoplasma gondii* infection to modulate host metabolism (i.e., an increase in serum triglycerides and liver metabolic dysfunction was measured) [23]. In cancer cells, the interaction between CCL5 and CCR5 increased the expression of glucose transporters, glucose uptake, glycolysis and subsequent ATP production [21]. In diabetic mice, blocking of the CCR5 receptor increased LDL and blood triglyceride levels [24]. These CCR5-influenced metabolic changes justify a metabolomics-based investigation of the chemokine receptor.

One of the requirements of metabolomics-based analysis is that a homogenous sample set be analysed so as to make conclusive remarks about the test variable. As part of future work, a metabolomics approach can therefore be used to study those parameters identified in this review to skew CCR5-based findings. By including or excluding these parameters in a metabolomics investigation, CCR5-specific conclusions can be made. Metabolomics makes use of highly sensitive and selective techniques [195]. To date, several different analytical platforms exist, yet there is no one analytical platform which can detect and measure all of the metabolites present within a sample. This is thought to be due to the diversity of the molecules as well as the different concentrations in which they are present. Nonetheless, several metabolomics approaches exist. Untargeted metabolomics approaches investigate the metabolic profile of samples in a global, unbiased manner and the data obtained can give a comprehensive overview of the metabolism. This approach is followed when there is no prior knowledge regarding the metabolites present in the sample and the method is therefore not focused on a specific molecule or class of molecules [196,197]. In contrast, a targeted approach focuses on a specific class of compounds to inform on biochemical pathways of interest. Using any of the aforementioned metabolomics approaches, new insights can be discovered regarding the altered metabolism, as effected through CCR5-induced immune changes.

From the articles reviewed here which report on metabolic parameters [21,22,23,24,61,62,63,64,66,72,73,103,173], a general trend regarding CCR5 in disease is that it impacts mainly lipid levels, cholesterol and the glycolysis pathway. Future work should therefore analyse samples displaying differential CCR5 status, using untargeted and targeted metabolomics approaches. Untargeted analysis will uncover metabolic changes not previously associated with CCR5 whilst targeted approaches will expand on the affected pathways already known to be impacted by CCR5 and further uncover mechanisms regarding the functioning of the molecule.

The metabolomics approach followed will be guided by the aim of the study which will in turn influence the choice of the biological sample and analytical platform. Sample choice is generally made based on ease of access. Biofluids that can be obtained non-invasively are typically the first choice to use and often includes urine. Furthermore, the use of blood is also a popular choice since its collection is minimally invasive. Muscle or tissue biopsies are generally hard to come by, which makes them less popular for research purposes. In addition to clinical samples which may be scarce, primary cell cultures or established cell lines can also be used for analysis. Cell lines are more homogenous in make-up and response and their environment is more controlled, hence less variation will be built into models used to better characterise CCR5. More conclusive remarks can thus be made regarding the role of CCR5 in the absence and/or presence of disease.

Preparation of the sample will ensure that it is compatible with a specific analytical platform. This may include but is not limited to: adding an internal standard; performing metabolite extraction and derivatization; or adding a buffer and after which the sample is subjected to the analytical platform for metabolomics analysis.

To conclude, chemokines and chemokine receptors such as CCR5 play a pivotal role in health and disease. Mutations present in genes encoding for these protein molecules can impact on immune and metabolic profiles and therefore influence disease susceptibility and progression rates. The metabolic changes can be particularly measured through metabolomics. This cascading impact of CCR5 on various biosystems is summarised in Figure 1. While the role of CCR5 in the context of HIV-1 infection is more defined, its role in other diseases remains contradictory. In an article published by Klein [53], the author also highlights the contrasting role(s) of CCR5 in diseases. However, our knowledge of CCR5 has increased in the last ten years. In addition, technological advancements make it possible to investigate CCR5 in more depth. This review therefore provides a more in-depth explanation for the role(s) of CCR5 in disease, and highlights metabolomics as a tool with which to clarify the contrasting role(s) of CCR5. Metabolic and immune profiling of samples presenting differential CCR5 status stand to further improve our understanding of this molecule.

## Figures and Tables

**Table:** (**A**)

Reference		[61]	[62]	[63]	[64]	[65]	[66]	[67]	[68]	[69]	[70]
**Category of Disease**		CVD	CVD	CVD	CVD	CVD	CVD	CVD	CVD	CVD	CVD
**Disease**		AS	CHD	CAD	CAD	CAD	CAD	CAD	CM	CAD	CAD
**Population**		Mixed	Caucasian	Caucasian	Greek	German; Caucasian	Indian	Bruneck	German	Hungarian	Mixed
**Model**		Meta-analysis	Blood	Blood	Blood	Blood	Blood	Blood	Blood	Blood	Meta-analysis
**CCR5∆32 frequency**	**Patients**	↑ ^Asian^					↑				
	**No difference**	X ^Mixed^	X	X	X	X			X		X
	**Controls**										
**CCR5 levels**	**Patients/test subject/sample**										
	**No difference**										
	**Controls**										
**Genotype**	**Homozygous CCR5∆32**	X ^Asian^	X	X		X		X	X		
	**Heterozygous CCR5∆32**	X ^Asian^	X	X		X		X	X	X	
	**Absent CCR5∆32**		X ^Px^		X ^a^		X ^a^			X ^Px,a^	
	**Homozygous WT**					X					
**Association/effect of CCR5∆32**	**Protective**			X ^a^				X	X ^M^	X ^a^	
	**No association/effect**	X ^S, Mixed^		X ^b^	X	X ^S^				X ^b,S^	X
	**Risk factor/non-protective**	X ^S,Asian^	X ^O^				X ^S^				
**Assay(s) used**	**PCR**		X	X	X	X	X	X	X	X	
	**RFLP**				X	X					
	**Sequencing**										
	**Immune-based**		X		X			X		X	
	**Microscopy**										
	**Spectroscopy**				X	X					
	**Flow Cytometry**										
	**Chromatography**										
	**MS**										
	**Other**	Meta-analysis									Meta-analysis
**Parameters**	**Immune**		X			X		X			
	**Metabolic**		X	X		X	X	X		X	

**Table:** (**B**)

Reference		[72]	[73]	[74]	[75]	[76]	[77]	[84]	[85]	[86]	[87]
**Category of Disease**		CVD	CVD	CVD	CVD	CVD	CVD	NS	NS	NS	NS
**Disease**		MI	MI	MI	ICAS	HT	HT	AD	AD	AD	AD
**Population**		Sicilian	Caucasian	Turkish	Italian	Caucasian	Caucasian	Spanish	Italian	Italian	Iranian
**Model**		Blood	Blood	Blood	Blood	Blood	Blood	Blood	Blood	Blood	Blood
**CCR5∆32 frequency**	**Patients**		↑ ^>60 years^	↑ ^a,b^		↑ ^a,b^					
	**No difference**				X		X	X	X	X	X
	**Controls**	↑	↑ ^<55 years^								
**CCR5 levels**	**Patients/test subject/sample**										
	**No difference**										
	**Controls**										
**Genotype**	**Homozygous CCR5∆32**	X		X	X	X	X				
	**Heterozygous CCR5∆32**	X	X	X	X	X	X				
	**Absent CCR5∆32**		X ^a^								
	**Homozygous WT**										
**Association/ effect of CCR5∆32**	**Protective**	X	X ^<55 years^								
	**No association/effect**				X		X	X	X^ P^	X	X
	**Risk factor/non-protective**		X ^>60 years^	X^ a,b^		X^ a,b^					
**Assay(s) used**	**PCR**	X	X	X	X	X	X	X	X	X	X
	**RFLP**			X		X				X	X
	**Sequencing**										
	**Immune-based**										
	**Microscopy**										
	**Spectroscopy**										
	**Flow Cytometry**										
	**Chromatography**										
	**MS**										
	**Other**										
**Parameters**	**Immune**										
	**Metabolic**			X							

**Table:** (**C**)

Reference		[88]	[90]	[91]	[92]	[93]	[94]	[95]	[96]	[97]	[100]	[101]
**Category of Disease**		NS	NS	NS	NS	NS	NS	NS	NS	NS	IS	IS
**Disease**		AD	PD	ms	ms	ms	ms	ms	ms	ms	Asthma	Asthma
**Population**		N/A	Spanish	Caucasian	Finland	Israeli	Dutch	Australian	Brazilian	Irish	Scottish	Scottish
**Model**		Mice	Blood	Tissue; Blood	Blood	Blood	Blood; Brain Tissue	Blood	Blood	Blood	Cells	Blood
**CCR5∆32 frequency**	**Patients**				↑ ^a^					↑		
	**No difference**		X				X	X	X	X		
	**Controls**											
**CCR5 levels**	**Patients/test subject/sample**	↓			↓							
	**No difference**											
	**Controls**											
**Genotype**	**Homozygous CCR5∆32**			X	X	X						
	**Heterozygous CCR5∆32**			X		X			X		X	
	**Absent CCR5∆32**								X^ a^		X^ Px^	X^Px,a,CA^
	**Homozygous WT**											
**Association/ effect of CCR5∆32**	**Protective**	X				X ^P,C^	X		X ^O,b^		X	X ^CA^
	**No association/effect**		X^ S,C^	X^ S^		X ^S^	X ^S,C^	X ^S^	X ^S,C^	X ^S,C,P^		
	**Risk factor/non-protective**			X ^C,M^	X^ S,C^					X ^O^		
**Assay(s) used**	**PCR**	X	X	X	X	X	X	X	X	X	X	X
	**RFLP**							X				
	**Sequencing**										X	
	**Immune-based**	X										
	**Microscopy**											
	**Spectroscopy**											
	**Flow Cytometry**				X							
	**Chromatography**											
	**MS**											
	**Other**											
**Parameters**	**Immune**	X										
	**Metabolic**											

**Table:** (**D**)

Reference		[103]	[104]	[105]	[106]	[24]	[110]	[111]	[112]	[113]	[114]	[115]
**Category of Disease**		IS	IS	IS	IS	IS	IS	IS	IS	IS	IS	IS
**Disease**		Asthma	Asthma	Asthma	T2DM	T2DM	T1DM	T1DM	T1DM	T1DM; T2DM	Asthma; T1DM	RA
**Population**		Mixed	Iranian	Polish	Dutch; Swedish	N/A	Italian	Hungarian	British	Estonian	Mixed	Brazilian
**Model**		Blood	Blood	Blood	Blood; Urine	Rats: Blood; Tissue	Blood	Blood	Blood	Blood	Meta-Analysis	Blood; Synovial fluid
**CCR5∆32 frequency**	**Patients**											
	**No difference**		X	X			X	X		X		X
	**Controls**		↑ ^b^									
**CCR5 levels**	**Patients/test subject/sample**					↓						↑ ^CCR5+cells^
	**No difference**											
	**Controls**											
**Genotype**	**Homozygous CCR5∆32**						X	X	X			
	**Heterozygous CCR5∆32**		X				X	X	X	X		X ^Px^
	**Absent CCR5∆32**		X^Px,a^									X^a^
	**Homozygous WT**											
**Association /effect of CCR5∆32**	**Protective**				X ^M^	X^ C^				X^ C,b^	X ^T1DM^	
	**No association/effect**	X	X	X			X ^S^	X	X	X ^S^	X ^Asthma^	X
	**Risk factor/non-protective**								X *			
**Assay(s) used**	**PCR**	X	X	X	X	X	X	X	X	X		X
	**RFLP**						X					
	**Sequencing**						X					
	**Immune-based**		X			X						
	**Microscopy**											
	**Spectroscopy**											
	**Flow Cytometry**											X
	**Chromatography**				X							
	**MS**											
	**Other**										Meta-analysis	
**Parameters**	**Immune**				X	X						
	**Metabolic**				X							

**Table:** (**E**)

Reference		[116]	[117]	[118]	[119]	[120]	[122]	[123]	[124]	[126]	[30]
**Category of Disease**		IS	IS	IS	IS	IS	IS	IS	IS	IS	ID
**Disease**		RA; JIA	RA	RA; SLE	RA	RA	SLE	SLE; LN	SLE	LN	AIDS
**Population**		Norwegian	Danish	Caucasian	New Zealand	German	Portuguese	Brazilian	Brazilian	Mixed	Mixed
**Model**		Blood	Blood	Blood	Blood	Blood	Blood	Blood	Blood	Meta-analysis	Meta-analysis
**CCR5∆32 frequency**	**Patients**				↓ ^b^	↑ *	↓ ^b^	↓ ^European;^ ↑^ African^	↑		↑ ^a,b,EU^
	**No difference**	X	X	X^ SLE^						X	
	**Controls**										
**CCR5 levels**	**Patients/test subject/sample**										
	**No difference**										
	**Controls**										
**Genotype**	**Homozygous CCR5∆32**	X	X^ C^		X	X			X		X
	**Heterozygous CCR5∆32**	X	X^ C^	X	X	X	X	X	X		X
	**Absent CCR5∆32**			X^ Px (RA),a^	X ^Px,a^		X^ Px,a^	X^ Px,a (European)^			
	**Homozygous WT**			X							
**Association /effect of CCR5∆32**	**Protective**		X ^C^	X ^RA,a^	X^a^	X ^S,C^	X	X ^SLE-European^			X ^S,P^
	**No association/effect**	X		X ^SLE^	X^ C^				X ^C^	X ^S,Caucasian,Asian^	
	**Risk factor/non-protective**							X ^LN African^	X ^S,O^	X^ African^	
**Assay(s) used**	**PCR**	X	X	X	X	X	X	X	X		
	**RFLP**										
	**Sequencing**										
	**Immune-based**					X			X		
	**Microscopy**										
	**Spectroscopy**										
	**Flow Cytometry**										
	**Chromatography**										
	**MS**										
	**Other**					X				Meta-analysis	Meta-analysis
**Parameters**	**Immune**					X					
	**Metabolic**										

**Table:** (**F**)

Reference		[31]	[33]	[37]	[128]	[129]	[130]	[131]	[132]	[133]	[134]
**Category of Disease**		ID	ID	ID	ID	ID	ID	ID	ID	ID	ID
**Disease**		AIDS	AIDS	AIDS	AIDS	AIDS	AIDS	AIDS	AIDS	AIDS	AIDS
**Population**		Caucasian	Black South African	Caucasian	European descent	Human	Italian	European descent	American	Caucasian	N/A
**Model**		Cells	Blood	Blood	Cells	Cells	Blood; cells	Blood	Blood	Blood	Blood
**CCR5∆32 frequency**	**Patients**	↓^ b^									
	**No difference**		X								
	**Controls**										
**CCR5 levels**	**Patients/test subject/sample**		↓			X ^NE^					
	**No difference**										
	**Controls**										
**Genotype**	**Homozygous CCR5∆32**	X		X	X	X	X	X	X	X	X
	**Heterozygous CCR5∆32**			X			X				
	**Absent CCR5∆32**	X ^Px,a^									
	**Homozygous WT**										
**Association /effect of CCR5∆32**	**Protective**	X	X	X ^S,P^	X	X					
	**No association/effect**										
	**Risk factor/non-protective**						X	X	X	X	X
**Assay(s) used**	**PCR**	X	X	X	X	X	X	X	X	X	X
	**RFLP**										
	**Sequencing**	X			X		X	X		X	X
	**Immune-based**		X			X		X	X		
	**Microscopy**					X					
	**Spectroscopy**				X						
	**Flow Cytometry**		X						X		
	**Chromatography**										
	**MS**										
	**Other**										
**Parameters**	**Immune**		X								
	**Metabolic**										

**Table:** (**G**)

Reference		[135]	[136]	[137]	[138]	[139]	[144]	[145]	[146]	[148]	[149]	[153]
**Category of Disease**		ID	ID	ID	ID	ID	ID	ID	ID	ID	ID	ID
**Disease**		AIDS	AIDS	WNF	WNF	WNF	HBV	HBV	HBV	HBV	HBV/HIV-1	HCV
**Population**		Caucasian	Caucasian	N/A	Caucasians	American	N/A	Iranian	Iranian	Caucasian	Brazilian	Mixed
**Model**		Blood	Blood	Mice	Blood; CSF	Meta-analysis	Blood; CD4^+^ cells	Blood; NK cells	Blood; CD8^+^ cells	Blood	Blood	Blood
**CCR5∆32 frequency**	**Patients**									↑^ Recovery^		
	**No difference**										X	
	**Controls**											
**CCR5 levels**	**Patients/test subject/sample**			↑	↓		↑ ^A^	↓	↓			↓ ^CCR5+cells^
	**No difference**											
	**Controls**											↑
**Genotype**	**Homozygous CCR5∆32**	X	X		X					X		
	**Heterozygous CCR5∆32**											
	**Absent CCR5∆32**								X ^Px^			X^ Px^
	**Homozygous WT**			X								X^ Px^
**Association/effect of CCR5∆32**	**Protective**			X ^M^			X			X^ C^	X^ HBV/HIV^	X
	**No association/effect**										X^S,HBV^	
	**Risk factor/ non-protective**	X	X		X	X		X	X			
**Assay(s) used**	**PCR**	X	X	X	X		X	X	X	X	X	X
	**RFLP**	X										
	**Sequencing**	X	X									
	**Immune-based**			X			X	X	X	X		X
	**Microscopy**											
	**Spectroscopy**											
	**Flow Cytometry**			X			X	X	X			X
	**Chromatography**											
	**MS**											
	**Other**					Meta-analysis						
**Parameters**	**Immune**			X			X					X
	**Metabolic**											

**Table:** (**H**)

Reference		[154]	[155]	[156]	[157]	[158]	[161]	[162]	[169]	[170]	[171]
**Category of Disease**		ID	ID	ID	ID	ID	ID	ID	ID	ID	ID
**Disease**		HCV	HCV	HCV	HCV	HCV	CD	CD	H1N1	H1N1	H1N1
**Population**		European	Caucasian	Jewish Israeli	Spanish	British	Mixed	Venezuelan	Mixed	Southern Europe	Brazilian admixed
**Model**		Tissue	Blood	Blood; Tissue	Blood; Tissue	Blood	Blood	Blood	Blood	Blood	Blood
**CCR5∆32 frequency**	**Patients**								↑^ b^	↓	
	**No difference**		X	X	X	X		X			X
	**Controls**										
**CCR5 levels**	**Patients/test subject/sample**						↑ ^Mild disease^; ↓ ^Severe disease^				
	**No difference**										
	**Controls**										
**Genotype**	**Homozygous CCR5∆32**	X									X
	**Heterozygous CCR5∆32**			X		X			X	X	X
	**Absent CCR5∆32**				X^ Px^			X			
	**Homozygous WT**										
**Association/effect of CCR5∆32**	**Protective**	X ^C^		X^ b^		X ^C^					
	**No association/effect**	X ^S^	X ^S,C^	X^ S,P^	X^ C^			X	X ^S^	X	X
	**Risk factor/non-protective**						X ^C^		X^ C^		
**Assay(s) used**	**PCR**	X	X	X	X	X		X	X	X	X
	**RFLP**		X								
	**Sequencing**										
	**Immune-based**					X		X			
	**Microscopy**										
	**Spectroscopy**										
	**Flow Cytometry**						X				
	**Chromatography**										
	**MS**										
	**Other**										
**Parameters**	**Immune**										
	**Metabolic**										

**Table:** (**I**)

Reference		[172]	[175]	[22]	[176]	[177]	[178]	[179]	[180]	[181]	[182]
**Category of Disease**		ID	Cancer	Cancer	Cancer	Cancer	Cancer	Cancer	Cancer	Cancer	Cancer
**Disease**		H1N1	General	General	General	BC	BC	BC	Carcinomas	PanC	CC
**Population**		Brazilian	N/A	Breast; tumour	Breast; tumour	Iranian	Mixed	N/A	Turkish	Mixed	Indian
**Model**		Tissue; cells	Mice	Cells	Cells	Blood	Meta-analysis	Human cells; Mice	Blood	Blood	Tissue
**CCR5∆32 frequency**	**Patients**								↑^ b,BC^		↑^ b^
	**No difference**	X									
	**Controls**										
**CCR5 levels**	**Patients/ test subject/ sample**		↓	↑	↓			↑			
	**No difference**										
	**Controls**										
**Genotype**	**Homozygous CCR5∆32**					X					
	**Heterozygous CCR5∆32**	X				X			X		X
	**Absent CCR5∆32**		X ^KO^								X^ Px,a^
	**Homozygous WT**	X									
**Association/effect of CCR5∆32**	**Protective**				X ^O,P^			X			
	**No association/effect**	X ^S.M^				X	X^ S^		X ^Carcinoma^	X ^P,C^	
	**Risk factor/ non-protective**		X	X					X ^BC^	X	X ^O^
**Assay(s) used**	**PCR**	X	X			X		X	X	X	X
	**RFLP**									X	
	**Sequencing**	X						X			
	**Immune-based**		X		X			X			
	**Microscopy**							X			
	**Spectroscopy**		X	X	X						
	**Flow Cytometry**		X	X	X			X			
	**Chromatography**			X							
	**MS**			X	X					X	
	**Other**						Meta-analysis				
**Parameters**	**Immune**										
	**Metabolic**			X	X						

**Table:** (**J**)

Reference		[183]	[184]	[185]	[186]	[187]	[188]	[189]	[190]	[191]	[192]
**Category of Disease**		Cancer	Cancer	Cancer	Cancer	Cancer	Cancer	Cancer	Cancer	Cancer	Cancer
**Disease**		CC; HPV	GallC	GasC	GasC	GasC	BlC	PC	PC	PC	AML
**Population**		Swedish	Indian	Japanese	Polish	Chinese	Turkish	Turkish	Brazilian	Australian	Iranian
**Model**		Tissue	Blood	Blood	Blood	Tissue; Cell line	Tissue	Blood	Blood	Blood	Blood
**CCR5∆32 frequency**	**Patients**	↑ ^(HPV+)^	↑ ^b^								
	**No difference**									X	
	**Controls**										↑^ b ^
**CCR5 levels**	**Patients/test subject/sample**			↑		↑					
	**No difference**										
	**Controls**										
**Genotype**	**Homozygous CCR5∆32**		X		X				X		
	**Heterozygous CCR5∆32**		X		X		X	X	X		
	**Absent CCR5∆32**							X			X^ Px^
	**Homozygous WT**										
**Association/effect of CCR5∆32**	**Protective**										
	**No association/effect**		X ^S^		X				X	X^ S^	X^ S^
	**Risk factor/non-protective**	X	X ^O^	X ^C^		X	X	X			
**Assay(s) used**	**PCR**	X	X		X	X	X	X	X	X	X
	**RFLP**		X				X	X	X		
	**Sequencing**	X									
	**Immune-based**			X	X	X					
	**Microscopy**										
	**Spectroscopy**										
	**Flow Cytometry**			X							
	**Chromatography**										
	**MS**										
	**Other**										
**Parameters**	**Immune**					X					
	**Metabolic**										

Table 1: The contrasting role of CCR5 in various diseases.↑ increased; ↓ decreased; ^Mixed^ Cohort contains individuals from various ethnicities; ^a^ Homozygous genotype; ^b^ heterozygous genotype; ^Px^ patient group; ^S^ susceptibility; ^O^ disease onset; ^P^ disease progression; ^C^ clinical course/severity of disease; ^M^ mortality; ^A^ acute; ^Ch^ chronic; ^BC^ breast cancer; ^KO^ CCR5 knockout mice; ^NE^ not expressed; ^WT^ wild type; ^CA^ childhood asthma; * sum effect, other CCR5 polymorphisms; ^EU^ exposed but uninfected; ^PC^ pancreatic cancer;. Abbreviations: PCR—polymerase chain reaction; RFLP—restriction fragment length polymorphism; MS—mass spectrometry; CVD—cardiovascular disease; AS—atherosclerosis; CHD—coronary heart disease; CAD—coronary artery disease; CM—cardiomyopathy; MI—myocardial infarction; ICAS—ischemic carotid artery stenosis; HT—hypertension; NS—nervous system disease; AD—Alzheimer’s disease; PD—Parkinson’s disease; ms—multiple sclerosis; IS—immune system disease; T2DM—type II diabetes mellitus; T1DM—type I diabetes mellitus; RA—rheumatoid arthritis; JIA—juvenile idiopathic arthritis; SLE—systemic lupus erythematosus; LN—lupus nephritis; ID—infectious disease;AIDS – acquired immunodeficiency syndrome; WNF—West Nile fever; HBV—hepatitis B virus; HCV—hepatitis C virus; CD—Chagas disease; PanC—pancreatic cancer; CC—cervical cancer; GallC—gallbladder cancer; GasC—gastric cancer; PC—prostate cancer; AML—acute myeloid leukemia.

## Abbreviations

CCR5	C-C chemokine receptor 5
kDa	kilodalton
CMI	Cell-mediated immunity
MIP1-α	Macrophage inflammatory protein one alpha
MIP1-β	Macrophage inflammatory protein one beta
RANTES	Regulated upon activation, normal T-cell expressed and secreted
CCL5	C-C chemokine ligand 5
MCP-2	Monocyte chemotactic protein two
Th1	T helper type 1
Th2	T helper type 2
ATP	Adenosine triphosphate
LDL	Low-density lipoprotein
HDL	High-density lipoprotein
∆32	32-base pair deletion
bp	Base pairs
WT	Wild type
HIV-1	Human immunodeficiency virus
AIDS	Acquired immunodeficiency syndrome
CCR5^+^	CCR5-positive
CRISPR	Clustered regularly interspaced short palindromic repeats
DNA	Deoxyribonucleic acid
UK	United Kingdom
CVD	Cardiovascular disease
MCP-1	Monocyte chemoattractant protein-1
CAD	Coronary artery disease
CRP	C-reactive protein
ELISA	Enzyme-linked immunosorbent assay
PCR	Polymerase chain reaction
HDL-C	High-density lipoprotein cholesterol
TG	Triglycerides
LDL-C	Low-density lipoprotein cholesterol
RFLP	Restriction fragment length polymorphism
MI	Myocardial infarction
ICAS	Ischemic carotid artery stenosis
AD	Alzheimer’s disease
PD	Parkinson’s disease
ms	Multiple sclerosis
RT-PCR	Reverse-transcription polymerase chain reaction
LOAD	Late-onset Alzheimer’s disease
mRNA	Messenger ribonucleic acid
IgE	Immunoglobulin E
T2DM	Type 2 diabetes mellitus
HbA1c	Haemoglobin A1c
T1DM	Type 1 diabetes mellitus
RA	Rheumatoid arthritis
RF	Rheumatoid factor
JIA	Juvenile idiopathic arthritis
SLE	Systemic lupus erythematosus
LN	Lupus nephritis
SDF-1	Stromal cell-derived factor one
WNV	West Nile virus
WNF	West Nile fever
HBV	Hepatitis B virus
NK	Natural killer
HCV	Hepatitis C virus
CCR5^-^	CCR5-negative
HPV	Human papillomavirus
HPV^+^	Human papillomavirus-positive
AML	Acute myeloid leukaemia
MS	Mass spectrometry
AS	Atherosclerosis
CHD	Coronary heart disease
CM	Cardiomyopathy
HT	Hypertension
NS	Nervous system
IS	Immune system
ID	Infectious disease
CD	Chagas disease
BC	Breast cancer
PanC	Pancreatic cancer
CC	Cervical cancer
GallC	Gallbladder cancer
GasC	Gastric cancer
PC	Prostate cancer
CCL3	C-C chemokine ligand 3
CCL4	C-C chemokine ligand 4
NP	Non-protective
PP	Partially protective
